# Molecular and Biochemical Analyses of CbCel9A/Cel48A, a Highly Secreted Multi-Modular Cellulase by *Caldicellulosiruptor bescii* during Growth on Crystalline Cellulose

**DOI:** 10.1371/journal.pone.0084172

**Published:** 2013-12-16

**Authors:** Zhuolin Yi, Xiaoyun Su, Vanessa Revindran, Roderick I. Mackie, Isaac Cann

**Affiliations:** 1 Energy Biosciences Institute, University of Illinois at Urbana-Champaign, Urbana, Illinois, United States of America; 2 Institute for Genomic Biology, University of Illinois at Urbana-Champaign, Urbana, Illinois, United States of America; 3 Department of Animal Sciences, University of Illinois at Urbana-Champaign, Urbana, Illinois, United States of America; 4 Department of Microbiology, University of Illinois at Urbana-Champaign, Urbana, Illinois, United States of America; Oak Ridge National Laboratory, United States of America

## Abstract

During growth on crystalline cellulose, the thermophilic bacterium *Caldicellulosiruptor bescii* secretes several cellulose-degrading enzymes. Among these enzymes is CelA (CbCel9A/Cel48A), which is reported as the most highly secreted cellulolytic enzyme in this bacterium. CbCel9A/Cel48A is a large multi-modular polypeptide, composed of an N-terminal catalytic glycoside hydrolase family 9 (GH9) module and a C-terminal GH48 catalytic module that are separated by a family 3c carbohydrate-binding module (CBM3c) and two identical CBM3bs. The wild-type CbCel9A/Cel48A and its truncational mutants were expressed in *Bacillus megaterium* and *Escherichia coli*, respectively. The wild-type polypeptide released twice the amount of glucose equivalents from Avicel than its truncational mutant that lacks the GH48 catalytic module. The truncational mutant harboring the GH9 module and the CBM3c was more thermostable than the wild-type protein, likely due to its compact structure. The main hydrolytic activity was present in the GH9 catalytic module, while the truncational mutant containing the GH48 module and the three CBMs was ineffective in degradation of either crystalline or amorphous cellulose. Interestingly, the GH9 and/or GH48 catalytic modules containing the CBM3bs form low-density particles during hydrolysis of crystalline cellulose. Moreover, TM3 (GH9/CBM3c) and TM2 (GH48 with three CBM3 modules) synergistically hydrolyze crystalline cellulose. Deletion of the CBM3bs or mutations that compromised their binding activity suggested that these CBMs are important during hydrolysis of crystalline cellulose. In agreement with this observation, seven of nine genes in a *C. bescii* gene cluster predicted to encode cellulose-degrading enzymes harbor CBM3bs. Based on our results, we hypothesize that *C. bescii* uses the GH48 module and the CBM3bs in CbCel9A/Cel48A to destabilize certain regions of crystalline cellulose for attack by the highly active GH9 module and other endoglucanases produced by this hyperthermophilic bacterium.

## Introduction

Cellulose, the most abundant renewable source of carbon and energy on our planet, is a linear chain of glucose units linked by β-1,4 glycosidic bonds. Cellulases are enzymes that can hydrolyze the large cellulose polymer into glucose or cello-oligosaccharides. In general, cellulases are classified into three groups, i.e. endoglucanases, exoglucanases (cellobiohydrolase) and β-glucosidases. Crystalline cellulose constitutes the core of cell-wall microfibrils, which are also highly recalcitrant to enzymatic hydrolysis [1]. 

*Caldicellulosiruptor bescii* formerly *Anaerocellum thermophilum*, belonging to the Firmicutes phylum and Thermoanaerobacterales Family III [2], is a highly thermophilic bacterium that degrades and utilizes crystalline cellulose and xylan, as well as more complex substrates such as untreated grasses and hardwood [3,4], at a temperature of 80°C [5]. Since the bacterium can utilize both cellulose and hemicelluloses at high temperatures, it is seen as a potential source of biocatalysts for plant cell wall deconstruction in the biofuel industry. The complete sequencing of the genome of *C. bescii* was recently accomplished, and analyses of the genome revealed a large gene cluster that encodes nine enzymes predicted to either degrade cellulose or hemicellulose [5,6]. When *C. bescii* is grown on crystalline cellulose, a large multi-modular enzyme (CelA) encoded by ORF1954 (GenBank accession number ACM60955) [7] is reported as the most highly secreted cellulolytic enzyme. The polypeptide represented 34.7% of total secreted proteins in a cellulose-based enrichment of *C. bescii* cellulases in the supernatant. The native form of the *C. bescii* CelA, which is composed of an N-terminal GH9 module and a C-terminal GH48 module separated by a CBM3c and two tandemly-linked CBM3bs of identical amino acid sequence, was purified for biochemical characterization [8]. The CBM3c and CBM3b were previously named C’ and C, respectively [8]. The polypeptide is designated CbCel9A/Cel48A in this study to denote the first GH9 and GH48 catalytic modules biochemically characterized from this bacterium. 

Based on the catalytic modules, CbCel9A/Cel48A is predicted to possess both endoglucanase and exoglucanase activities. A protein in *Caldicellulosiruptor obsidiansis* (GenBank accession number: ADL42953) has similar domain architecture to CbCel9A/CbCel48A. The two proteins in *C. bescii* and *C. obsidiansis* share 94% amino acid sequence identity, and it is reported that the *C. obsidiansis* ortholog is also one of the most highly secreted proteins during growth of *C. obsidiansis* on crystalline cellulose [7,9]. Besides these two *Caldicellulosiruptor* species, orthologs of CbCel9A/Cel48A with high amino acid sequence identities (96% and 94%, respectively) are found in two other *Caldicellulosiruptor* strains, i.e., ORF906 of *C. kronotskyensis* (GenBank accession number: ADQ45727) and ORF1238 of *C. saccharolyticus* (GenBank accession number: ABP66690). Therefore, these proteins likely play a critical role in cellulose hydrolysis and fermentation by these bacteria. 

The GH9 catalytic domain is an (α/α)_6_ barrel fold which contains an open active site cleft that holds six glucopyranose-binding subsites from -4 to +2 [10]. The biochemically characterized GH9 modules exhibit different enzymatic activities [11], including endo-β-1,4-glucanase, cellobiohydrolase, 1,4-β-D-glucan glucohydrolase, β-glucosidase, exo-β-glucosaminidase, and processive endoglucanase activities. All of the processive endoglucanases biochemically characterized in this family contain a CBM3c attached to the C-terminus of the GH9 catalytic module [10,12]. While CBM3c is commonly linked to the GH9 catalytic module, CBM3b is associated with diverse catalytic modules.

The structures of GH48 catalytic modules indicate that their active sites are a composite of both open clefts and closed tunnels [13,14,15,16]. The GH48 modules likely have 10 sugar-binding subsites, 7 of which are in the closed tunnel region and the remaining 3 in the open cleft. Members of the GH48 family also have diverse enzymatic activities, including cellobiohydrolase [17,18,19,20,21], endo-β-1,4-glucanase [17], chitinase [22], and processive endocellulase activities [23]. 

In this study, the wild-type and the truncational mutants of the *C. bescii* CbCel9A/Cel48A were biochemically characterized to determine which activities are present in the two catalytic modules. It was anticipated that dissecting the functional activities of the different modules in CbCel9A/Cel48A will provide insights into its potential use in facilitating plant cell wall deconstruction at high temperatures for application in the biofuel industry. 

## Results and Discussion

### Expression and purification of CbCel9A/Cel48A wild-type and its truncational mutants

**Figure 1:**
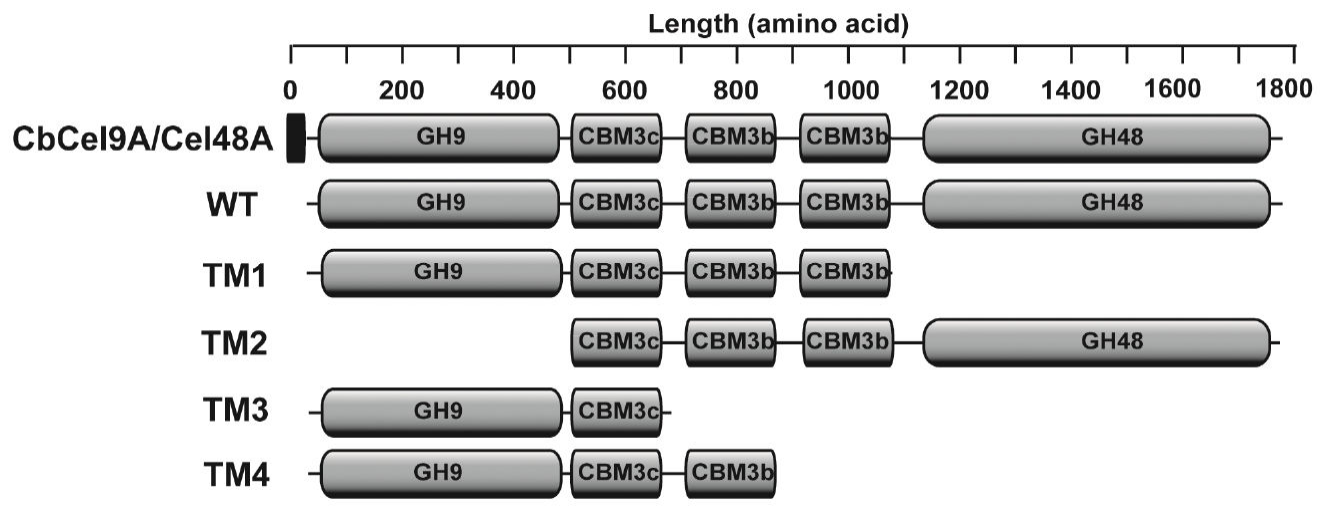
Schematic representation of CbCel9A/Cel48A wild-type (WT) and its truncational mutants. The signal peptide is represented by the filled rectangle. GH9: glycoside hydrolase family 9 module; GH48: glycoside hydrolase family 8 module; CBM3b: carbohydrate-binding module family 3 type B; CBM3c: carbohydrate-binding module family 3 type C.

The CbCel9A/Cel48A of *C. bescii* was previously purified in its native form and demonstrated to function as a cellulase that degrades the model crystalline cellulose Avicel [8]. The enzyme was also reported to be highly expressed during growth of *C. bescii* on crystalline cellulose [7]. CbCel9A/Cel48A is a large polypeptide with five modules (Figure 1). Thus, to gain insight into the contribution of the different modules to its enzymatic activity, we made several truncational mutants. Expression of the wild-type CbCel9A/Cel48A and TM2 (composed of the three CBM3s and the GH48 module) failed in *E. coli* strains that have been previously used to express genes from *C. bescii* in our laboratory [11,24,25,26]. In contrast, the same polypeptides were successfully expressed in *Bacillus megaterium*. The truncational mutants TM1 (composed of the GH9 module and the three CBM3s) and TM3 (composed of the GH9 and the CBM3c) were successfully expressed in *E. coli*. All recombinant proteins were purified to near homogeneity, and their apparent molecular masses corresponded well to their calculated values (results not shown). The native form of CbCel9A/Cel48A exhibited an apparent molecular mass of 230 kDa, which is about 40 kDa larger than the calculated value and also the molecular mass estimated through SDS-PAGE for the recombinant form of the polypeptide (~190 kDa, Figure S1 in File S2). This difference in the molecular mass of CbCel9A/Cel48A suggests either glycosylation or other post-translational modifications of the native form of the polypeptide in *C. bescii* [8].

### Optimum pH, Specific Activities, Kinetic Parameters and Thermostability of CbCel9A/Cel48A Wild-Type and Its truncational Mutants

The optimum pH for the WT protein, using PASC as the substrate, was between pH 5.5 and pH 6.5, and this is similar to the result obtained by Zverlov et al. [8]. The optimum pH observed for TM1 was pH 5.0 (Figure S2 in File S2). TM2 showed very low activity in each of the conditions tested and its optimum pH was observed to be around pH 5.0 (Figure S2 in File S2). The specific activity of CbCel9A/Cel48A WT was 22.8±1.3 µM glucose equivalents min^-1^ µM^-1^ enzyme on Avicel (Table 1), which was lower than that of its native form (calculated to be 55 µM glucose equivalents min^-1^ µM^-1^ enzyme) [8]. The lower activity of the recombinant WT protein may come from its lack of post-translational modification that likely occur for this polypeptide in *C. bescii*. Deletion of either catalytic module from the WT protein (TM1 and TM2) and also deletion of the two CBM3bs from TM1 (to create TM3) decreased the specific activities of the enzyme on crystalline cellulose, i.e., Avicel and filter paper (Table 1). This observation is similar to that described by Zverlov et al., in which a truncated derivative CelA’ (a rough equivalent of TM4 deduced by its calculated molecular mass, Figure 1) showed lower specific activity on the model crystalline cellulose Avicel compared with CelA (i.e., CbCel9A/Cel48A wild-type in this study) [8]. Thus, deletion of the GH48 module decreased the specific activity of the enzyme by about half (10.2±1.0 µM glucose equivalents min^-1^ µM^-1^ enzyme on Avicel). Deletion of the two CBM3bs (TM3) further impaired the enzymatic activity, with only 15% of the WT specific activity retained on Avicel (Table 1). Similar trends were observed when filter paper was used as the substrate. Removal of the GH9 module (TM2) dramatically decreased the specific activity of the enzyme on Avicel and filter paper, with only about 10% of the WT activity retained for TM2 on both substrates. 

**Table 1:** Kinetic parameters of CbCel9A/Cel48A wild-type and its truncational mutants on cellulosic substrates^***a***^.

Enzyme	PASC**^*b*^**			Avicel (µM glucose equivalents/min/µM enzyme)	Filter paper (µM glucose equivalents/min/µM enzyme)
	*k*_cat_ (s^-1^)	*K*_*m*_ (mg ml^-1^)	*k*_cat_/*K*_m_ (s^-1^ ml mg^-1^)		
WT	2.0±0.3	2.6±1.0	0.8±0.3	22.8±1.3	13.1±0.3
TM1	1.6±0.2	1.8±0.6	0.9±0.3	10.2±1.0	7.0±1.8
TM2	0.2±0.0	1.5±0.6	0.1±0.0	2.2±0.1	1.3±0.2
TM3	5.1±0.3	2.3±0.3	2.2±0.3	3.5±0.7	4.5±0.6

^a^ The reactions were performed in a 50 mM sodium-citrate buffer with 150 mM NaCl (pH6.0) at 75°C.

^b^ PASC: phosphoric acid swollen cellulose.

The kinetic parameters of the WT protein and its truncational mutants were estimated using the amorphous cellulose PASC as the substrate. None of the truncational mutants showed a large difference in *K*_m_ compared with the WT protein. Interestingly, TM1 showed a similar catalytic efficiency (*k*_cat_/*K*_m_) to that of WT protein. TM2 exhibited a large decrease (by 87%) in its catalytic efficiency compared with that of the WT protein. In contrast, the TM3 mutant had an increased catalytic efficiency, which was 2.8-fold higher than that of the WT protein (Table 1). The results indicated that all components of CbCel9A/Cel48A play important roles in hydrolysis of crystalline cellulose. In addition, the GH9/CBM3c modules make the most contribution to the hydrolysis of the non-crystalline cellulose PASC. The analyses above, together with previous data [8] showed that the GH9 module in CbCel9A/Cel48A is responsible for the endoglucanase activity of the polypeptide. 

A sequential deletion of the GH48 catalytic module and one or two of the CBM3bs from the WT protein or deletion of the GH9 module impacted the thermostability. Interestingly, the recombinant WT protein was less thermostable than its truncational mutants at 70°C (Figure S3 in File S2). The residual activity of WT was 49.0±2.7% after treatment at 70°C for 20 h. This is lower than those of TM1, TM2, TM3, and TM4, which were 76.5±4.1%, 89.2±3.8%, 127.9±4.3%, and 88.6±13.2%, respectively. At 75°C, the residual activities of WT, TM1, TM2, TM3, and TM4 after 20 h treatment was 41.6±4.3%, 35.9±1.5%, 35.1±3.8%, 74.5±3.9%, and 49.5±2.2%, respectively. At 80°C, the residual activities of TM1, TM2, and TM4 were all lower than that of WT (33.1±4.5%), whereas TM3 retained 45.0±1.9% activity (Figure S3 in File S2). Therefore, TM3 (GH9-CBM3c) has a higher thermostability than the WT protein and the other mutants at all temperatures investigated, and this is likely due to the compact structure of the GH9/CBM3c, which appear to have evolved as a functional unit. Similar results were observed for a truncational mutant, TM3, of CbCel9B/Man5A from the same bacterium [11]. The residual activity of TM3 after heating at 70°C for 20 hours was found to be 127.9±4.3%. Although unexpected, it is known that certain thermostable proteins show higher functional activities after heat treatment, which may be due to their proper folding at higher temperatures [27]. Thus, the increased hydrolytic activity observed in the truncational mutant TM3, after heating, may in part be explained by this characteristic observed in some thermophilic proteins.

The CBM3c is commonly observed as fused to a GH9 module, and this type of CBM is supposed to convey a single cellulose chain into the GH9 active site [10,11,12,28,29]. There might be a specific non-covalent interaction between the GH9 module and CBM3c [30], and the absence of the CBM3c results in a large loss of enzymatic activity of GH9 module [11,12,28,31]. The co-crystal structure of the GH9/CBM3c in complex with cello-oligosaccharide has been reported [10,32]; however, the absence of ligand binding by the CBM3c in the structure has hindered a detailed understanding of its role in the function of the GH9 catalytic module. 

### Enzymatic activities of CbCel9A/Cel48A wild-type and its truncational mutants on cellulosic substrates

**Figure 2:**
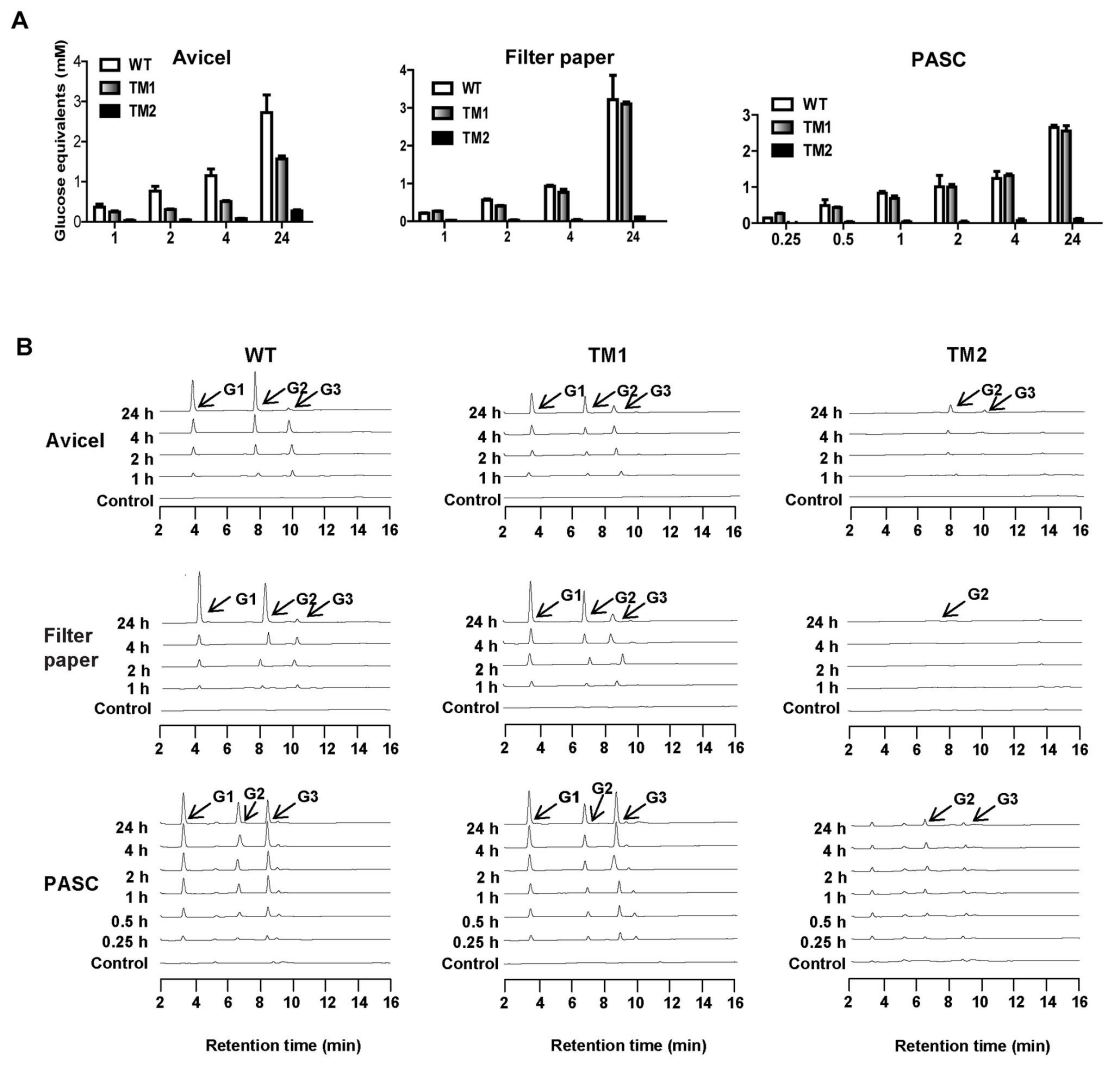
Time course hydrolysis of Avicel, Filter paper, and PASC by CbCel9A/Cel48A and its truncational mutants analyzed by a reducing sugar assay (A) and HPLC analysis (B). The reactions were performed in citrate buffer (pH 6.0) at 75°C. Substrates were 10 mg ml^-1^ of Avicel or 16 discs of Whatman No. 1 filter paper (0.6 cm in diameter) or 2.5 mg ml^-1^ of PASC. Enzyme concentrations on Avicel and filter paper were 0.5 μM and 0.2 μM on PASC, or 0 μM for the control.

Avicel, filter paper, and PASC were used as model cellulose substrates of different structures and degrees of recalcitrance in evaluating the activities of the WT protein and its truncational mutants. On Avicel, little to no hydrolysis was observed for TM2 in the reducing sugar assay. In contrast, TM1 released significant amounts of glucose equivalents. The WT protein released about twice the amounts of glucose equivalents observed for TM1 at all times of sampling (1, 2, 4, and 24 hours). However, on filter paper and PASC, while the TM2 mutant still released almost no glucose equivalents, the WT protein and TM1 released comparable amounts of glucose equivalents (Figure 2A).

The end products of hydrolysis by the WT protein and the TM1 mutant on all substrates were quite similar (Figure 2B), with the major constituents being glucose, cellobiose, and cellotriose. On PASC, WT and TM1 released similar amounts of cellotriose, cellobiose and glucose at all times of sampling (1, 2, 4, and 24 hours), and the amounts of cellotriose was comparable to those of cellobiose and glucose, which were different for WT and TM1 on crystalline cellulose (Figure 2B, and Table S2 in File S1). On Avicel, TM1 released smaller amounts of cellobiose, glucose and cellotriose than WT at all times, except for the amounts of cellotriose at 24 hours of sampling. Compared to WT, after 24 hours incubation on Avicel, TM1 released more cellotriose. On Filter paper, compared with WT, TM1 released similar amounts of cellobiose, glucose and cellotriose, and also enhanced the relative amount of cellotriose after 24 hours incubation. On all 3 substrates, TM2 only released weak signals of end products (Figure 2B and Table S2 in File S1). The TM3 mutant was derived from TM1 by deleting the two CBM3bs, and it was found to be less effective in releasing glucose equivalents from both Avicel and filter paper than TM1. However, TM3 produced similar amounts of glucose equivalents from PASC to that of the WT protein (Table S2 in File S1). This observation suggests that the CBM3bs play an important role in hydrolysis of the crystalline cellulose but not in hydrolysis of non-crystalline cellulose.

**Figure 3:**
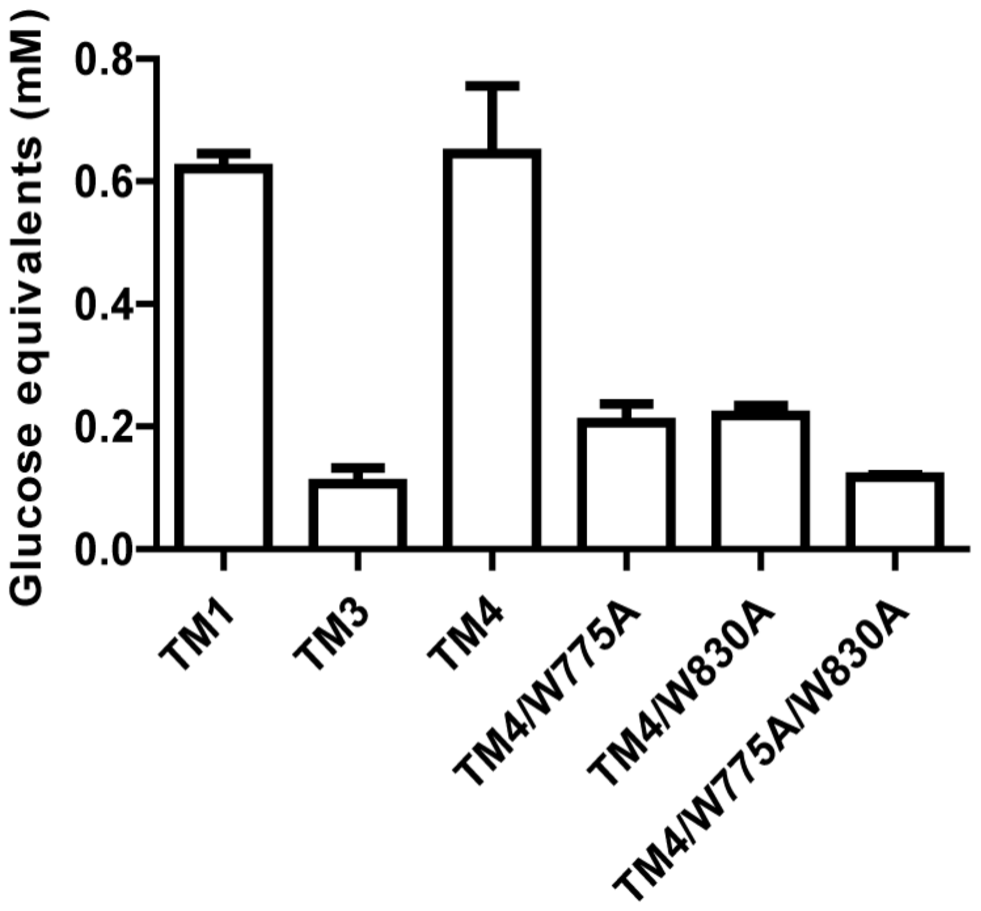
Hydrolysis of Avicel by TM1, TM3, TM4 and site-directed mutants of TM4. All of TM4 and its site mutants (TM4/W775A, TM4/W830A, and TM4/W775A/W830A) were expressed in *E. coli*. The experiment was performed by incubating 0.2 µM of the purified TM1, TM3, TM4 or the site-directed mutants of TM4 respectively with 10 mg ml^-1^ Avicel in citrate buffer (pH 6.0) at 75°C for 13 h.

The observed role of the CBM3bs in the hydrolysis of cellulosic substrates is consistent with reports in the literature. The members of CBM3b bind strongly to crystalline cellulose [11,12,31], and the two CBM3bs from CbCel9A/Cel48A are identical to the CBM3bs from the *C. bescii* CbCel9B/Man5A (ORF1952, GenBank accession number ACM60953), which we earlier showed to bind tightly to crystalline cellulose [11]. To obtain more detailed information about the role of the CBM3bs, we made TM4, a truncational mutant composed of the GH9 module, CBM3c, and one CBM3b (Figure 1). This mutant showed similar hydrolytic capacity on Avicel as the TM1 mutant (Figure 3). Thus, a tandem repeat of CBM3b in the same polypeptide did not improve the hydrolysis of the crystalline cellulose (Avicel). A similar observation was made for CbCel9B/Man5A [11]. 

In the CBM3b of CbCel9A/Cel48A, two tryptophan residues (W775 and W830) were predicted to form the cellulose-binding platform, based on the structure of a homologous CBM3b from *Acetivibrio cellulolyticus* [33]. To further confirm the observed role of the CBM3bs in the hydrolysis of crystalline cellulose, we mutated these tryptophan residues of TM4 individually or simultaneously to alanine. The single mutant (W775A or W830A) and the double mutant (W775A/W830A) of TM4 showed dramatic decreases in hydrolytic activity on crystalline cellulose (Figure 3). In fact, the mutations led to comparable activities to those of TM3 on crystalline cellulose. We hypothesize that the impaired activity is due to failure of the mutant proteins to bind to crystalline cellulose through the CBM3b. Interestingly, identical or highly similar (with an amino acid sequence identity >98%) CBM3bs are found in seven of the nine enzymes encoded in the gene cluster in which CbCel9A/Cel48A is located on the genome of *C. bescii* [11]. Thus, the CBM3b in the proteins in the gene cluster likely assist in the hydrolysis of crystalline cellulose and therefore are an important component of the plant cell wall degrading machinery of *C. bescii*. 

### Hydrolysis of cello-oligosaccharides by CbCel9A/Cel48A wild-type and its truncational mutants

**Figure 4:**
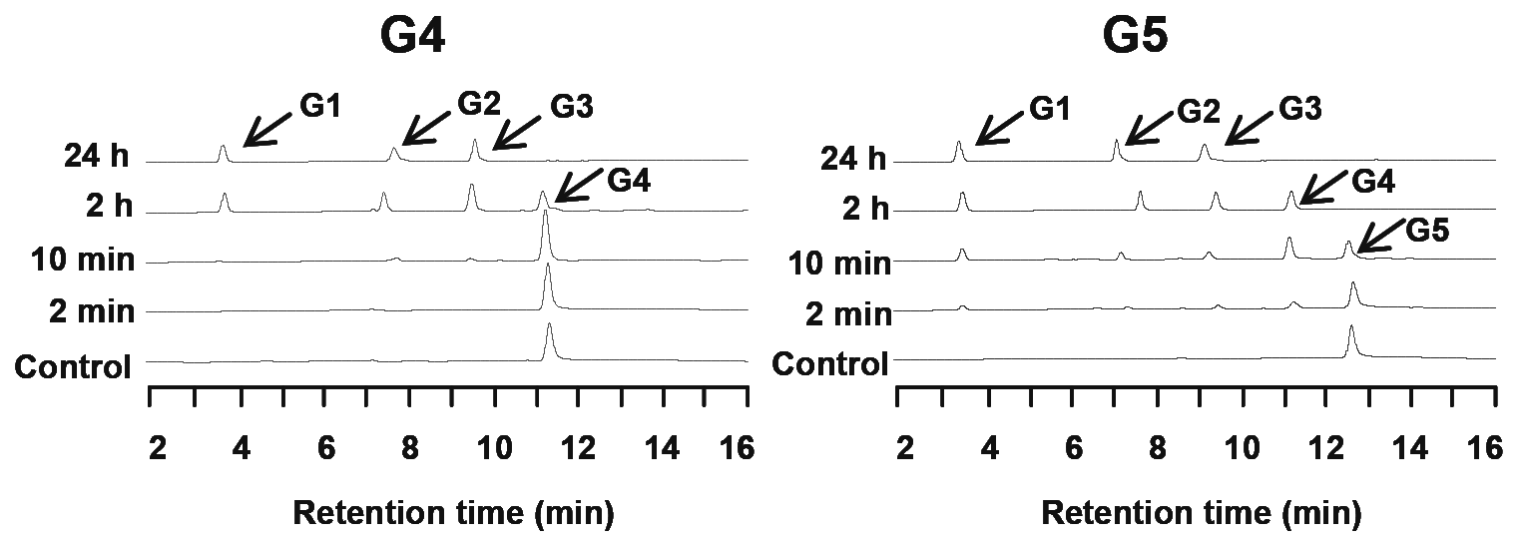
Time course hydrolysis of cellotetraose (G4) and cellopentaose (G5) by CbCel9A/Cel48A. The reactions were performed by incubating 0µM (for the control) or 0.2 µM of each enzyme with 2.5 mg ml^-1^ of cellotetraose (G4) or cellopentaose (G5) in a citrate buffer (pH 6.0) at 75°C for 24 h. Samples were taken at different time points and analyzed using an HPLC method.

The WT protein converted cello-oligosaccharides (cellotetraose and cellopentaose) to glucose, cellobiose, and cellotriose after 24 h of incubation (Figure 4), and this result is similar to observations for the native protein [8]. The TM1 mutant is a deletion of the GH48 module from the WT protein, and the TM3 mutant is a derivative of TM1 with the two CBM3bs deleted (Figure 1). The TM1 and TM3 mutants showed the same hydrolytic patterns of cellotetraose and cellopentaose as the WT recombinant protein (Table S3 in File S1). Since the hydrolytic patterns of TM1 and TM3 were similar to that of WT, it is concluded that the CBM3bs and GH48 module are not critical to hydrolysis of the cello-oligosaccharides investigated. 

After 24 h of incubation on cellotetraose and cellopentaose, TM2 produced only very small amounts of cellobiose and cellotriose (Table S3 in File S1). The pattern of the end products was similar to the results reported for other GH48 modules [19,20,23,34,35]. Taking into account the hydrolysis of cellulose by TM2, with major cellobiose and minor cellotriose (no glucose) as its end products (Figure 2), this pattern suggests that the GH48 module in CbCel9A/Cel48A is a cellobiohydrolase and confirms an earlier prediction by others [8]. A typical cellobiohydrolase releases cellobiose as the major end product with minor amounts of cellotriose and glucose [36,37]. Therefore the recombinant form of the Cel48A module of the polypeptide under study is an unusual cellobiohydrolase, since TM2 showed low activities on all tested cellulosic substrates and also did not release glucose as an end-product after long incubation with substrates (Figure 2B, Table S2 and Table S3 in File S1).

Based on an amino acid sequence alignment with other members of the GH48 family with known three-dimensional structures (Figure S4 and Figure S5 in File S3), we inferred that the GH48 of CbCel9A/Cel48A has 10 sugar-binding subsites [13,14,15,16]. As a result, this catalytic module would prefer long cello-oligosaccharides of 10 or more sugar moieties as substrate. We were unable to test this hypothesis, as we could not readily access oligosaccharides of the right degree of polymerization. We look forward to testing our hypothesis in future experiments. Amino acid sequence analysis showed that the GH48 modules of CbCel9A/Cel48A and the processive endoglucanase CcCel48F of *Clostridium cellulolyticum* [23] share about 63% identity. While the latter enzyme mainly releases G4 and small amounts of G2, G3, G5, and G6 after a short period of incubation (10 mins) with PASC [23], TM2 only released G2 and trace amounts of G3 after 15 mins of incubation with PASC. A similar observation has been made for the *Thermobifida fusca* TfCel48A [34]. The amino acid sequence alignment also showed that the GH48 module of CbCel9A/Cel48A has the same candidate acid/base residues (Figure S4 in File S3). In addition, most of the other residues involved in the interaction between the catalytic module of CcCel48A with cello-oligosaccharides are also conserved in CbCel9A/Cel48A. The only exception is the Arg211 residue of the GH48 in CbCel9A/Cel48A. While this residue is conserved in TfCel48A (Arg219), it is replaced by a lysine (Lys224) in CcCel48F (Figure S4 in File S3). It has been proposed that Lys224 of CcCel48F makes flexible contact with the sugars [16]. Thus the subtle differences, including the one at this site, are possible reasons for the difference in hydrolytic pattern observed with the GH48 module of CbCel9A/Cel48A. This hypothesis can be examined through site directed mutagenesis in the future.

### Reconstituting the wild-type activity using TM1 and TM2

**Figure 5:**
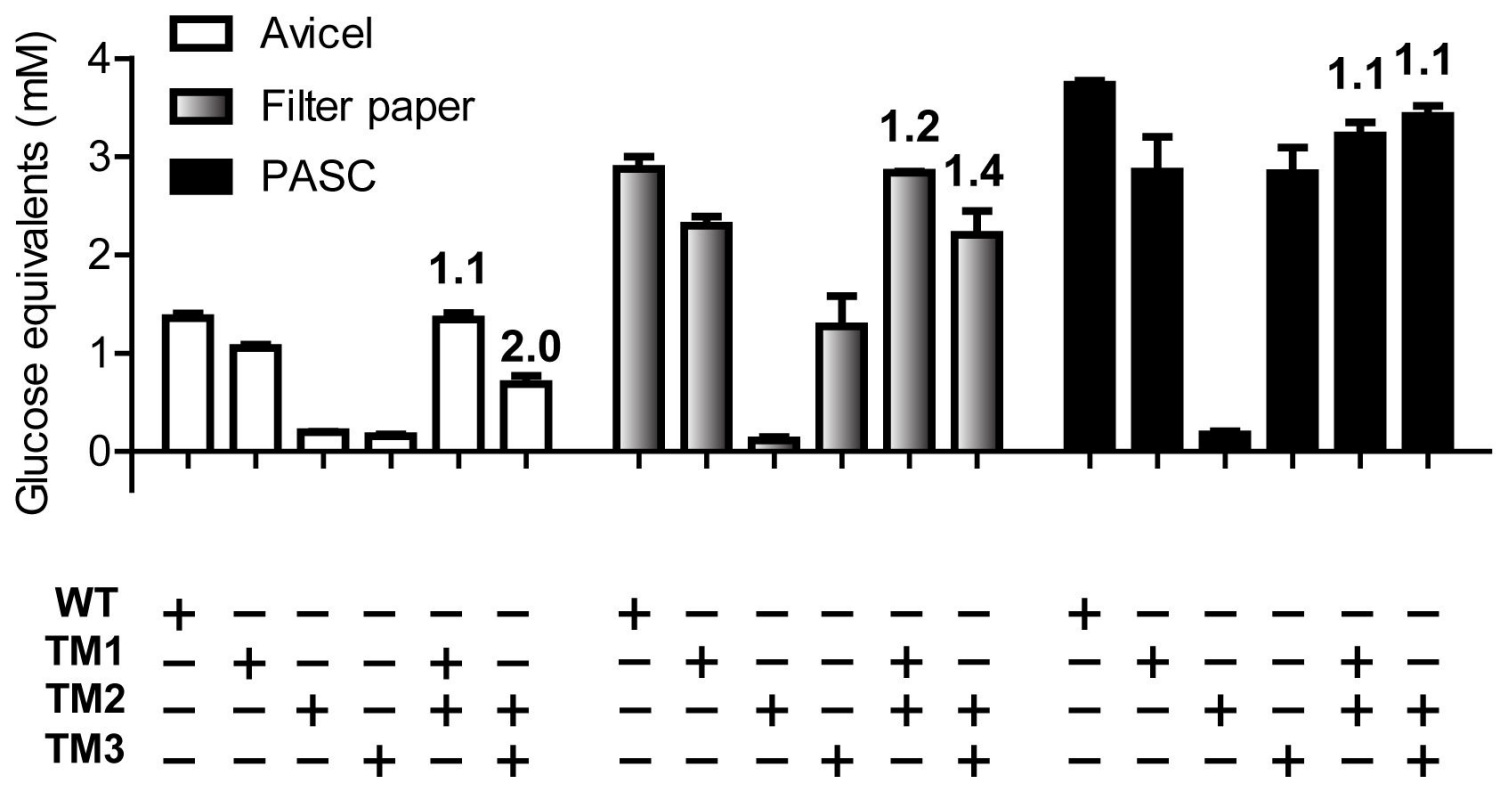
Hydrolysis of Avicel, Filter paper and PASC by CbCel9A/Cel48A, its truncational mutants, and their binary combinations as analyzed by a reducing sugar assay. The experiments were carried out in citrate buffer (pH 6.0) at 75°C for 16 h by incubating 0.4 µM of each enzyme with 10 mg ml^-1^ of Avicel or with 16 discs of Whatman No. 1 filter paper (0.6 cm in diameter) or by incubating 0.2 µM of each enzyme with 2.5 mg ml^-1^ of PASC as the substrate. The numbers on top of the columns represent the degrees of synergy calculated as described in *Materials* and *Methods*.

Experiments were carried out to determine whether the activity in the WT protein can be reconstituted with the two truncational mutants. While a TM1/TM2 co-incubation could achieve similar degrees of hydrolysis on Avicel to that of the WT protein, a TM2/TM3 co-incubation only reached about half of the glucose equivalents produced by either the WT (Figure 5) or the TM1/TM2 co-incubation. These results suggest the presence of intramolecular synergism in CbCel9A/Cel48A, an observation also made by Zverlov et al. [8]. Similar results were obtained when filter paper was used as the substrate. The TM3 mutant performed better on filter paper than Avicel, whereas its activity was similar to that of TM1 on PASC (Figure 5). These observations were confirmed by the HPLC analysis as shown in Figure S6 in File S3.

The importance of the CBMs in reconstituting the wild-type activity was further examined. As mentioned above, the GH9 module of TM3 together with the CBM3c has been proposed to hydrolyze single cellulose chains efficiently. PASC is an amorphous (non-crystalline) cellulose with many available single cellulose chains. Therefore, PASC should be easily bound and hydrolyzed by TM3. Note that even on the surface of crystalline celluloses, such as Avicel and filter paper, there are still single cellulose chains based on their fractions of β-glucosidic bonds accessible to cellulase attack [36,38]. We hypothesize that, without the CBM3b, TM3 binds less efficiently to crystalline celluloses, thereby releasing less glucose equivalents from Avicel and filter paper than TM1 (Figure 5). Although filter paper and Avicel have similar crystallinity index values, filter paper has a higher maximum protein absorption capacity and a higher proportion of β-glycosidic bonds available to cellulase cleavage [38,39], and this may explain why TM3 releases more glucose equivalents from filter paper than from Avicel (Figure 5). TM1, with both CBM3b and CBM3c, more efficiently binds and catalyzes the hydrolysis of crystalline celluloses, therefore releasing more glucose equivalents than TM3 on such substrates. 

The data in Figure 5 shows that the GH48 module in TM2 is important for the hydrolysis of crystalline cellulose by the GH9 module. However, on its own, TM2 releases only a small amount of end products as shown in Figure S6 in File S3. Most members of the GH48 family also show very low hydrolytic activity on Avicel, PASC, filter paper and cello-oligosaccharides as substrates [17,19,20,21,23,34,35,40]. It is, therefore, surprising to observe that enzymes belonging to the GH48 family are common and highly expressed in cellulolytic bacteria [7,9,19,20,23,41,42]. The high expression suggests an important role of GH48 enzymes in cellulose hydrolysis [43], although GH48 enzymes do not seem to exhibit an advantage over other cellobiohydrolases in terms of releasing glucose equivalents [34]. Thus, it is reasonable to postulate that GH48 enzymes might have an important, although yet to be completely elucidated, function in cellulose hydrolysis.

Although the GH48 module of TM2 only exhibited weak activity in the standard reducing sugar assay, we noticed that low-density Avicel-derived particles floated on the surface of the reaction mixture and also stuck to the inner walls of the Eppendorf tubes and pipet tips when WT, TM1, and TM2, but not TM3, were used to hydrolyze Avicel (data not shown). Upon observing SEM images, we discovered that the surface of the Avicel treated with WT, TM1 and TM2 enzymes showed some surface disruption compared with that of the non-treated Avicel or Avicel treated with TM3 (Figure S7 in File S4). We propose that the CBM3bs cause disruption of crystalline cellulose, making it more accessible to the GH9 catalytic module. However, the GH48 module could also be involved in such loosening activity, since it is predicted to have a long active site composed of 10 or more sugar binding subsites. We further hypothesize that the multi-modular structure of CbCel9A/Cel48A with tandem CBM3bs, in addition to the long active site of GH48, allows the protein to bind and destabilize the crystalline cellulose, making it more accessible to the GH9 catalytic module. The newly generated single cellulose chains then become substrates for hydrolysis by the GH9 module, with its accessory CBM3c feeding the strands into the active site. In agreement with this hypothesis, TM3 (the derivative of TM1 with the two CBM3bs deleted) showed high degrees of synergy (DOS) of 2.0 and 1.4 with TM2 in hydrolyzing Avicel and filter paper, respectively (Figure 5). In contrast, much lower DOSs of 1.1 and 1.2 respectively, were found between TM1 and TM2 on Avicel and filter paper (Figure 5). A possible explanation may be that the synergy was masked because the CBM3bs within TM1 also loosened the cellulose. Further experiments are needed to test our hypothesis. A cartoon depicting our hypothesis of synergistic degradation of crystalline cellulose by the GH9 and the GH48 modules is presented in Figure S8 in File S4.

### Effects of varying concentrations of truncational mutants on cellulose hydrolysis

**Figure 6:**
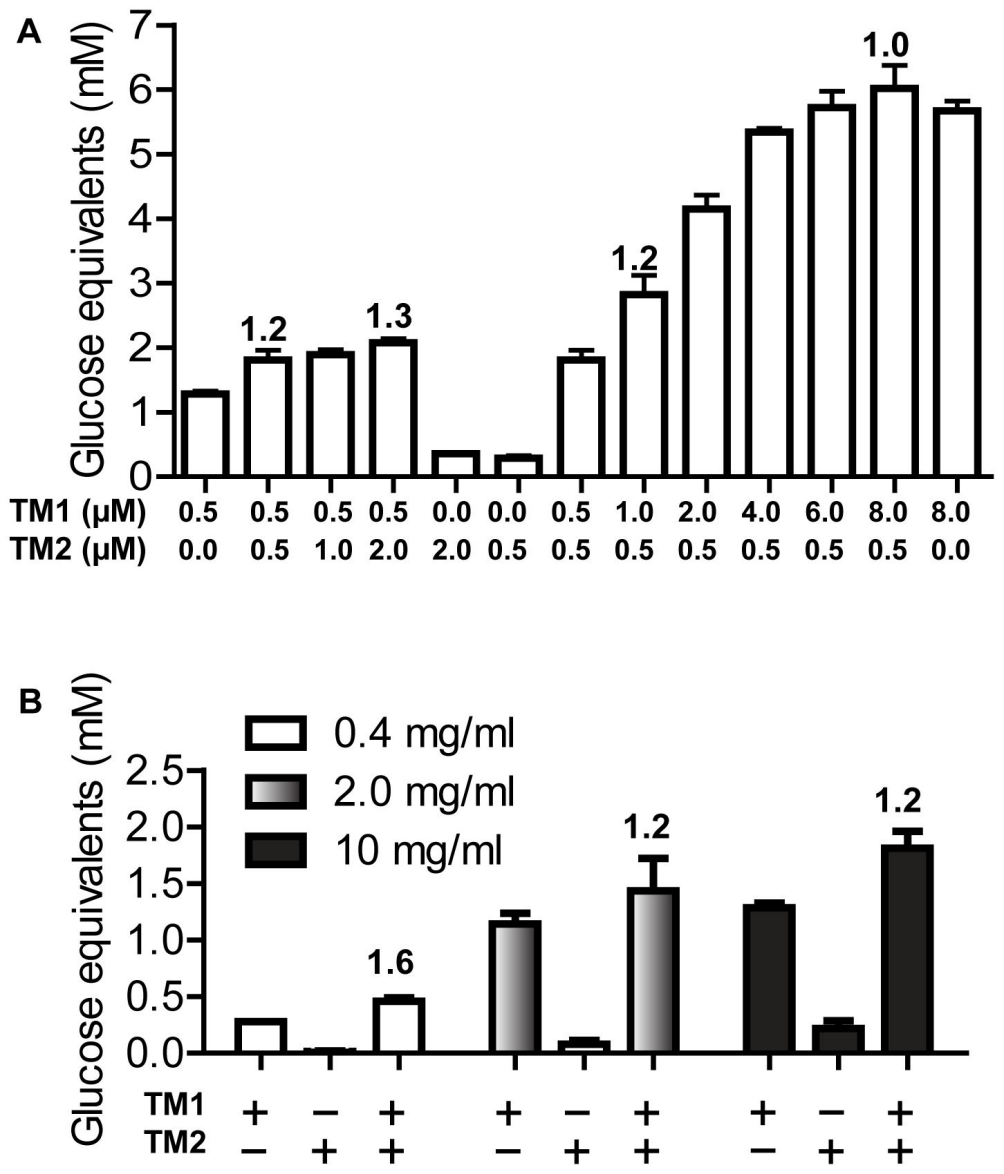
Synergistic action of TM1 and TM2 in hydrolysis of Avicel. The experiments were carried out in a citrate buffer (pH 6.0) at 75°C for 18 h. In (A), the substrate concentration was constant (10 mg ml^-1^ of Avicel), and the enzymes are combinations of TM1 and TM2 at different concentrations as shown in the figure. In (B), the concentrations of Avicel are different (0.4, 2, 10 mg ml^-1^) and the enzyme concentration (0.5 µM) is constant for TM1 and TM2. The numbers on top of the columns in Panels A and B represent the degrees of synergy calculated as described in *Materials* and *Methods*.

An interest in studying the recombinant CbCel9ACel48A is its potential application in plant cell wall polysaccharide hydrolysis in the biofuel industry. Thus, experiments were carried out to determine whether by varying the concentration of one of the truncational mutants or the WT protein, we can increase hydrolysis of the crystalline cellulose Avicel. When the concentration of TM1 was held constant, quadrupling the concentration of TM2 resulted in only a slight increase in glucose equivalents, although the increase seemed to indicate moderate levels of synergism (1.2 - 1.3). In another experiment, the TM2 mutant was held constant while increasing the concentration of TM1. In this case, an increasing release of glucose equivalents was observed with each increase in the concentration of TM1 (up to 8 μM) in the reaction mixture. However, there was no synergism for these reactions, and the increased yields of glucose equivalents appeared to be only due to the increased concentration of TM1 in the reaction mixture (Figure 6A). As synergism was observed when TM1 was held constant and TM2 was varied, this again suggested that TM2 produced end products that could serve as substrates for the GH9 module in TM1. 

The potential of the two truncational mutants to act synergistically was further examined by adding equal concentrations of enzymes (0.5 µM) into varied concentrations of Avicel. At a lower substrate concentration (0.4 mg ml^-1^ of Avicel), a high synergy (1.6) was observed (Figure 6B). However, as the substrate concentration was increased (2.0 mg ml^-1^ and 10 mg ml^-1^), there was a reduction in synergistic activity to 1.2 (Figure 6B). Based on our hypothesis above, addition of more TM2 in the reaction would result in more flexible single cellulose chains, which would subsequently lead to a slight increase in the DOS between TM1 and TM2. A lower starting concentration of crystalline cellulose or Avicel was concomitant with lower numbers of available single cellulose chains, which should lead to a larger increase of DOS between TM1 and TM2. This phenomenon may substantiate the hypothesis that an intramolecular synergistic effect exists between the catalytic domains of CbCel9A/Cel48A. Since both TM1 and TM2 have two CBM3bs that enable each truncational mutant to be independently adsorbed onto cellulosic substrates, it is likely that the two polypeptides exist in close proximity to each other at a lower substrate concentration. Cel9I and Cel48Y from *Clostridium thermocellum* were also reported to hydrolyze crystalline cellulose synergistically [21]. The CelY (containing a GH48 catalytic module) and CelZ (containing a GH9 catalytic module) from *Clostridium stercorarium* were assembled as a fusion protein in a study, where both intermolecular and intramolecular synergistic effects were observed between the two catalytic modules [44]. However, there are two significant differences between CbCel9A/Cel48A and the fusion protein CelYZ. First, the domain structure of CbCel9A/Cel48A is GH9-CBM3c-CBM3b-CBM3b-GH48, while that of CelYZ fusion protein is GH48-B-C-GH9-C’ (where B is a CBM_X2, C is CBM3b, and C’ is CBM3c). It is not known whether adjusting the order of the modules in the chimeric protein to that of CbCel9A/Cel48A will affect the activity of the fusion protein. Second, the biochemical property of the GH48 in CbCel9A/Cel48A is much different from that of the *C. stercorarium* CelY. In CbCel9A/Cel48A, the TM2 harboring the GH48 catalytic module has weak activity in hydrolyzing both crystalline cellulose and non-crystalline cellulose. However, the GH48 of *C. stercorarium* CelY is very active in hydrolyzing the non-crystalline cellulose PASC, although it is similarly weak at digesting the model crystalline cellulose Avicel [18]. 

### Effects of CbCel9A/Cel48A on enzymatic activity of *C. bescii* endoglucanases

**Figure 7:**
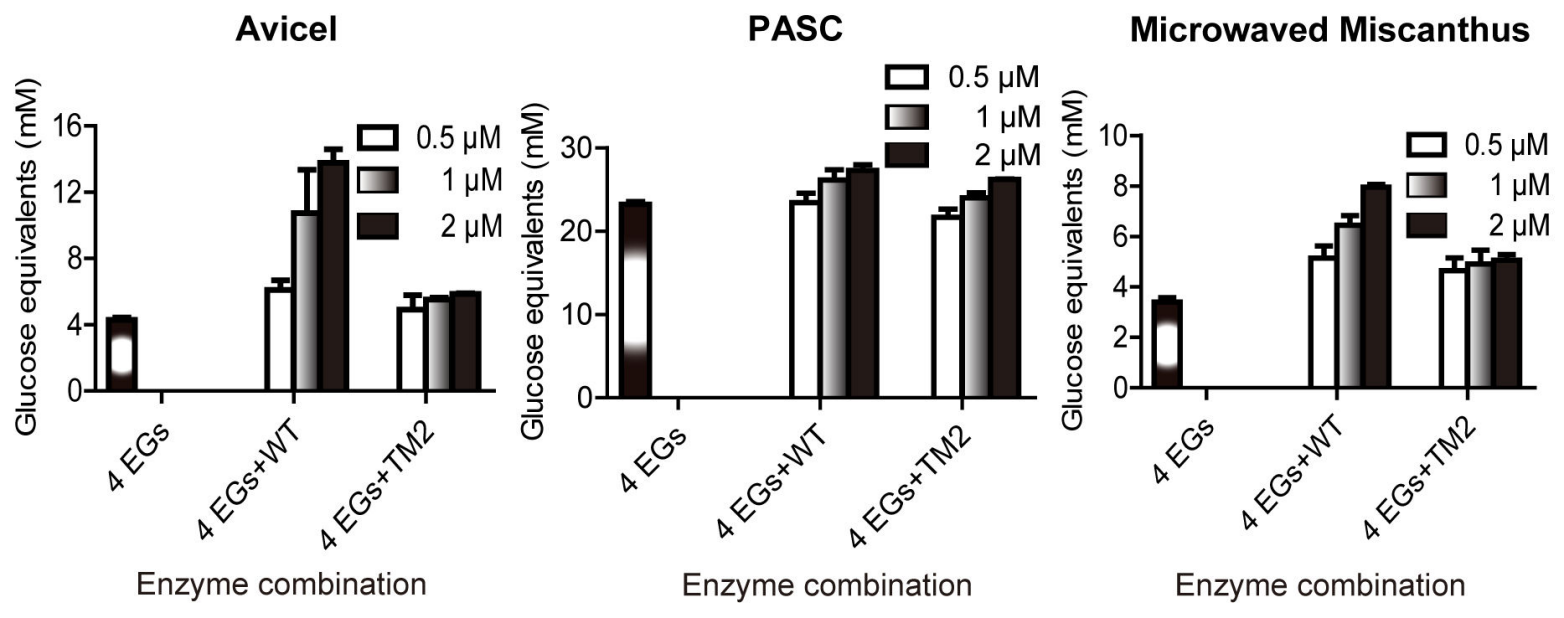
Effect of cellulose hydrolysis by four endoglucanases in the presence of either CbCel9A/Cel48A WT or the TM2 mutant. 4EGs: a mixture of four recombinant endoglucanses from *C. bescii* (CbMan5B/Cel44A-TM2, CbCel9B/Man5A-TM1, CbMan5C/Cel5A-TM2, and CbCel5B-TM1). The reactions were carried out in a citrate buffer (pH 5.5) at 70°C for 16 h, by incubating different concentrations (0, 0.5, 1.0, and 2.0 µM) of WT or TM2 with 5 mg ml^-1^ of Avicel, 5 mg ml^-1^ of PASC, or 10 mg ml^-1^ of Miscanthus, in the presence of the four endoglucanases (4EGs, each at 0.5 µM).

CbCel9A/Cel48A is the most highly expressed cellulase during growth of *C. bescii* on crystalline cellulose. To mimic this condition, we increased the concentration of the WT protein while holding the concentration of four other recombinant endoglucanases from *C. bescii* [26] constant during hydrolysis of Avicel. Interestingly, nearly 14 mM glucose equivalents were released from Avicel when only 2 µM of the WT protein was added to the endoglucanase mixture (Figure 7). A similar observation was made with the more complex substrate, pre-treated Miscanthus, a bioenergy feedstock. However, this effect was not seen with the non-crystalline cellulose PASC as substrate. These observations were also confirmed by the HPLC analysis (Figure S9 in File S4). Since CbCel9A/Cel48A is the highest cellulase secreted by *C. bescii*, its capacity to release large amounts of end products with the four endoglucanases (each at 0.5 µM) supports its important role in acquisition of nutrients by *C. bescii* from complex polysaccharides in the environment. Therefore, the recombinant WT protein may be of utility in preparing enzyme cocktails for deconstruction of plant cell wall polysaccharides into its component sugars, an essential step in biofuel production.

Members of the GH48 family have been shown through genetics to be central in crystalline cellulose degradation. For example, the CtCel48S deletion mutant of *C. thermocellum* exhibited a reduced rate of Avicel hydrolysis and also yield of cell mass, when the bacterium was grown on Avicel [45]. The reduced growth of *C. thermocellum* was explained by several HYPOTHESIS 1 of the hypotheses suggested that deletion of *cel48S* reduces the average length of the cello-oligomers in the end products of hydrolysis by the *C. thermocellum* cellulosome and this leads to a decrease in ATPs generated by the cell from the end products [46]. In the present report, the GH48 module of CbCel9A/Cel48A is proposed to aid in loosening crystalline cellulose to generate easily accessible cellulose chains. These less ordered forms or regions of cellulose serve as substrates for attack and hydrolysis by the other enzymes in the cellulolytic system of *C. bescii*. We look forward to carrying out further experiments that examine this hypothesis.

## Materials and Methods

### Materials

*Escherichia coli* DH10B strain (Life Technologies, Grand Island, NY) was used for gene cloning and plasmid maintenance throughout the study. *E. coli* BL21-CodonPlus (DE3) RIPL strain (Stratagene, La Jolla, CA) and *Bacillus megaterium* WH320 (MoBiTec, Goettingen, Germany) were used for gene expression. Isopropyl β-D-1-thiogalactopyranoside (IPTG) was purchased from Gold Biotechnology (St. Louis, MO). Cello-oligosaccharides (G2-G6) were obtained from Megazyme (Wicklow, Ireland). Avicel, xylose, and glucose were purchased from Sigma-Aldrich (St. Louis, MO). The *LA Taq* DNA Polymerase was from Takara (Shiga, Japan). The QIAprep Spin Miniprep Kit was obtained from Qiagen (Valencia, CA). The Talon metal affinity resin was purchased from Clontech Laboratories, Inc. (Mountain View, CA), and the Amicon Ultra centrifugal filters were obtained from Millipore (Billerica, MA). All other reagents were purchased from Fisher Scientific (Pittsburgh, PA).

### Gene Cloning and Expression

The complete genome sequence of *C. bescii* was analyzed using the RAST server [47] as previously reported [11]. The gene product of ORF1954 (CelA) was originally sequenced and characterized as a cellulase from the culture supernatant of *C. bescii* [8]. In the present study, CelA is designated CbCel9A/Cel48A based on the presence of two catalytic modules, i.e., GH9 and GH48, in the polypeptide. The GH9 and GH48 of CelA are the first members of their respective family characterized in this bacterium, hence the designation of the polypeptide under study as CbCel9A/Cel48A. The nucleotide sequences encoding the wild-type (WT) and four truncational mutants (TM1, TM2, TM3 and TM4; shown in Figure 1) were amplified with *LA Taq* DNA Polymerase using *C. bescii* genomic DNA as the template. The primers used for the PCR amplifications are shown in Table S1 in File S1. The TM4 site-directed mutants (W775A, W830A and W775A/W830A) were generated using the Quikchange Lightning Multi Site-directed mutagenesis kit according to the instructions of the manufacturer (Agilent Technologies, Santa Clara, CA). The plasmid encoding TM4 was used as the template, and the mutagenic primers used are listed in Table S1 in File S1. 

The PCR products were separated by agarose gel electrophoresis, and the DNA bands corresponding to the sequences encoding TM1, TM3, TM4 and all three site-directed mutants of TM4 were excised from the gel, purified, and treated with the exonuclease activity of T4 DNA polymerase according to the instructions of the manufacturer (Novagen, San Diego, CA). Each T4 DNA polymerase-treated DNA was then annealed to pET-46 Ek/LIC vector (Novagen, San Diego, CA) and transformed into *E. coli* DH10B. The recombinant *E. coli* cells were selected on lysogeny broth [48,49] (LB, containing 10 g l^-1^ tryptone, 5 g l^-1^ yeast extract, and 5 g l^-1^ sodium chloride) agar plates supplemented with ampicillin at a concentration of 100 μg ml^-1^. Single colonies were picked and cultured overnight in LB medium containing the same antibiotic. The plasmid DNA in each cultured colony was extracted (Qiagen mini-prep kit) for DNA sequencing (W. M. Keck Center for Comparative and Functional Genomics at University of Illinois at Urbana-Champaign) to confirm the integrity of the DNA insert. The recombinant plasmids containing the correct DNA inserts (TM1-, TM3-, TM4- and its site-directed mutants encoding nucleotide sequences) were transformed into *E. coli* BL21-CodonPlus (DE3) RIPL strain (Stratagene, La Jolla, CA) for gene expression. After an overnight incubation on LB agar plates supplemented with 100 µg ml^-1^ ampicillin and 50 µg ml^-1^ chloramphenicol at 37°C, a single colony was picked and inoculated into 10 ml of LB medium supplemented with the same antibiotics and incubated at 37°C with shaking (250 rpm) for 6 h. The pre-culture was inoculated into 1 liter LB medium supplemented with both antibiotics (ampicillin and chloramphenicol) at the concentrations described above and cultured at 37°C with vigorous shaking until the OD_600_ (optical density at 600 nm) reached 0.3. The temperature was shifted to 16°C and IPTG was added at a final concentration of 0.1 mM to induce gene expression. After another 16 hours of culturing, the cells were harvested by centrifugation (4,651 × *g* for 20 min at 4°C), and the cell pellet was re-suspended in 40 ml of lysis buffer (50 mM Tris-HCl, 300 mM NaCl, pH 7.5), and the cells were ruptured by two passages through an EmulsiFlex C-3 cell homogenizer from Avestin (Ottawa, Canada) to release their contents. The ruptured cells were centrifuged at 12,857 × g for 20 min at 4°C to separate the supernatant from the cell debris. The recombinant TM1, TM3, TM4 and the site-directed mutants of TM4 were purified by metal affinity chromatography as described below.

The PCR products for the WT polypeptide and its TM2 derivative were also separated on an agarose gel, purified, and ligated into a pGEM-T Vector (Promega, Madison, WI), followed by transformation into *E. coli* DH10B competent cells. The transformants were selected on LB agar plates supplemented with ampicillin at a concentration of 100 µg ml^-1^. The recombinant plasmids were isolated from the transformants and the DNA inserts in the plasmids were sequenced to confirm the integrity of the coding sequence. The plasmids were restriction digested with BglII and Acc65I to release the DNA inserts, which were then cloned into a BglII/Acc65I*-*digested *E. coli*-*Bacillus megaterium* shuttle vector pC-His1622 (MoBiTec, Goettingen, Germany). The ligated products were transformed into the *E. coli* DH10B competent cells and selected on LB agar plates supplemented with ampicillin (100 µg ml^-1^). After verifying the integrity of the DNA inserts by nucleotide sequencing, the recombinant pC-His1622 plasmids with the correct DNA inserts were transformed into *B. megaterium* WH320 protoplasts according to the instructions of the manufacturer (MoBiTec, Goettingen, Germany). Single *B. megaterium* colonies were picked from LB agar plates supplemented with 10 µg ml^-1^ of tetracycline, inoculated into 10 ml LB medium supplemented with the same antibiotic at 37°C, and cultured with shaking at 250 rpm for 8 h. The 10-ml pre-culture was inoculated into 1 liter LB medium supplemented with tetracycline (10 µg ml^-1^) and the culture was incubated at 37°C until the OD_600_ reached 0.3. Xylose was then added to the culture at a final concentration of 0.2% (w/v) to induce the expression of recombinant proteins. After another 12 h of culturing at 37°C, the cells were harvested by centrifugation (4,651 × *g* for 20 min at 4°C), re-suspended in 80 ml of lysis buffer, and homogenized by sonication with the settings of 60 repetitions of 15 sec sonication on and 15 sec off at an amplitude of 30% using a Sonic Dismembrator with a tip of 17.5 mm in diameter (Model 500, Fisher Scientific, Pittsburgh, PA). 

### Purification of proteins

Since *C. bescii* is a hyperthermophilic bacterium, its proteins are expected to be thermostable. Therefore, the cell lysates were heat-treated at 55°C for 20 min and clarified by centrifugation at 12,857 × *g* for 20 min at 4°C. After another heat treatment at 65°C for 20 min, the cell-lysates were centrifuged again under the same conditions, and the supernatants were collected. The supernatants were applied to the metal affinity resin (cobalt charged resin - Clontech, Mountain View, CA) and washed several times with lysis buffer. The bound proteins were eluted with binding buffer (50 mM Tris-HCl, 300 mM NaCl, pH 7.5) supplemented with 250 mM imidazole. All eluted fractions were examined by 12% sodium dodecyl sulfate-polyacrylamide gel electrophoresis (SDS-PAGE) followed by staining with Coomassie brilliant blue G-250. The fractions containing the desired proteins were pooled, the proteins were concentrated, and the buffer was changed to a protein storage buffer (50 mM Tris-HCl, 150 mM NaCl, pH 7.5) with Amicon Ultra-15 centrifugal filters. The protein concentrations were determined as described previously [50] using a NanoDrop 1000 (Thermo Fisher Scientific Inc., Waltham, MA) with the following calculated extinction coefficients: 550,765 M^-1^ cm^-1^, 304,610 M^-1^ cm^-1^, 393,865 M^-1^ cm^-1^, 189,790 M^-1^ cm^-1^ and 248,690 M^-1^ cm^-1^ for WT, TM1, TM2, TM3 and TM4 respectively.

### Determination of pH optima

The pH optima of CbCel9A/Cel48A WT and its truncational mutants (TM1 and TM2) were determined by incubating the enzymes (400 nM for WT and TM1 and 600 nM for TM2) with PASC (1 mg ml^-1^) for 1 hour in a citrate buffer ranging from pH 3.0 to pH 6.0 and in a phosphate buffer ranging from pH 5.5 to pH 8.0 at 75°C. The released glucose equivalents were measured using the 4-hydroxybenzoic acid hydrazine (*p*HBAH, Sigma-Aldrich, St. Louis, MO) method [51]. For negative controls, reaction mixtures without the enzymes were incubated for the same period as the reactions with the enzymes and analyzed for release of glucose equivalents. The released glucose equivalents from the negative controls were subtracted from each reaction to obtain the amount of glucose equivalents released by the enzyme. 

### Enzyme assays

The specific activities of the CbCel9A/Cel48A WT and its truncational mutants were determined using crystalline celluloses as substrates. Different concentrations of enzymes were incubated with 10 mg ml^-1^ of Avicel or 16 discs of Whatman No. 1 filter paper (0.6 cm in diameter) in a citrate buffer (50 mM sodium-citrate, 150 mM NaCl, pH 6.0) and shaken end-over-end for 90 min at 75°C. A control was prepared by incubating the substrates without any enzymes under the same reaction conditions. At different time intervals, samples were taken and centrifuged, and the released soluble glucose equivalents were measured by *p*HBAH method. The specific activities were determined within the range where the slopes of released glucose equivalents versus time were linear.

An appropriate concentration of each enzyme was incubated with a range of concentrations of phosphoric acid swollen cellulose (PASC) at 75°C in a citrate buffer (pH 6.0). The initial velocities were determined and plotted against the substrate concentrations, and the kinetic parameters were estimated by fitting the data to the Michaelis-Menten equation using a non-linear regression method (GraphPad Prism v5.01 Software, San, Diego, CA).

### Thermostability assay

The thermostability of CbCel9A/Cel48A and its truncational mutants were determined by incubating the enzymes (2 µM) in a citrate buffer (pH 6.0) at 70°C, 75°C, and 80°C (WT, TM1, TM2, TM3, and TM4) on a Veriti 96-well thermal cycler (Applied Biosystems, Carlsbad, CA). Samples were taken at different time points of 0 h, 1 h, 2 h, 4 h, 8 h, and 24 h. The residual enzymatic activity was determined by incubating 200 nM enzymes (except 400 nM for TM2) with PASC (1 mg ml^-1^) at 75°C in a citrate buffer (pH 6.0) for 1 hour (except 2 hours for TM2). 

### Time course hydrolysis of cellulosic substrates and cello-oligosaccharides

The reactions were carried out in citrate buffer (pH 6.0) at 75°C. For the analysis of hydrolysis of Avicel and filter paper, 0.5 µM of CbCel9A/Cel48A or its truncational mutants was incubated with 10 mg ml^-1^ of Avicel or 16 discs of Whatman No. 1 filter paper (0.6 cm in diameter). In the case of PASC, 0.2 µM of each enzyme was incubated with 2.5 mg ml^-1^ PASC. Aliquots of the reaction mixtures were taken at different time intervals (0.25 h, 0.5 h, 1 h, 2 h, 4 h, and 24 h), and the released soluble glucose equivalents were analyzed by the *p*HBAH method. The reaction end products were further analyzed by high-performance anion-exchange chromatography with a pulsed amperometric detector (HPAEC-PAD), as described in our previous report [24]. Hydrolysis of cello-oligosaccharides was carried out by incubating 0.2 µM of each enzyme with 2.5 mg ml^-1^ of cellotetraose (G4) or cellopentaose (G5), respectively, and samples were taken at different time intervals (2 min, 10 min, 2 h, and 24 h) and analyzed by the HPAEC-PAD method. 

### Hydrolysis patterns of CbCel9A/Cel48A wild-type and its truncational mutants on cellulosic substrates

Each enzyme at a concentration of 0.4 µM was incubated with either 10 mg ml^-1^ of Avicel or 16 discs of Whatman No. 1 filter paper (0.6 cm in diameter) in citrate buffer (pH 6.0) at 75°C for 16 hours. In the hydrolysis of PASC, the enzymes were at a concentration of 0.2 µM, and the substrate was at 2.5 mg ml^-1^. The end products of the reactions were analyzed by both the *p*HBAH and the HPAEC-PAD methods. The degrees of synergy (DOS) were calculated by the following equation: DOS = (Glucose equivalents released by enzyme combination)/(Sum of glucose equivalents released by individual enzymes) [25].

### Synergistic activities of TM1 and TM2 on Avicel

The experiments were carried out in citrate buffer (pH 6.0) at 75°C for 18 h by incubating 10 mg ml^-1^ of Avicel with varying concentrations of TM1 (0.0-8.0 µM) and TM2 (0.0-2.0 µM) or by incubating different concentrations of Avicel (0.4, 2, and 10 mg ml^-1^) with a fixed concentration of enzymes (0.5 µM of TM1 and 0.5 µM of TM2). The released soluble glucose equivalents were determined with the *p*HBAH method. 

### Effect of incubating CbCel9A/Cel48A wild-type or TM2 in combination with four endoglucanases from *C. bescii*

Recombinant *C. bescii* endoglucanases including CbMan5B/Cel44A-TM2 (composed of two CBM3b modules and one GH44 catalytic module) [26], CbCel9B/Man5A-TM1 (composed of a GH9 catalytic module, one CBM3c, and two CBM3b modules, [11]), CbMan5C/Cel5A-TM2 (composed of three CBM3b modules and one GH5 catalytic module), and CbCel5B-TM1 (composed of a GH5 catalytic module and one CBM28 module) were purified as described previously [26]. The capacity of either CbCel9A/Cel48A or its TM2 truncation mutant to synergistically hydrolyze cellulosic substrates in presence of other *C. bescii* cellulases was determined by incubating each of the two enzymes in combination with the four cellulases. The experiments were carried out in citrate buffer (pH 5.5) at 70°C for 16 h. The enzyme concentration of each of the four previously described endoglucanases [26] was held constant at 0.5 µM in the reaction mixture while the concentrations of the WT or TM2 were varied (0, 0.5, 1.0, and 2.0 µM). The substrates used were 5 mg ml^-1^ of Avicel or PASC or 10 mg ml^-1^ of Miscanthus (pretreated as described previously [52]). The soluble products were analyzed by the *p*HBAH method and HPAEC-PAD. 

### Scanning electron microscopy (SEM)

An environmental scanning electron microscope with a field-emission electron gun (XL30 ESEM-FEG; Philips/FEI Co., Hillsboro OR), operated in HiVac mode at 5 kV, with a spot size of 2.1 nm, was used to image Avicel samples after 24 hours hydrolysis at 75°C by the WT protein or its truncational mutants. Samples were coated using a Denton Desk II TSC turbo-pumped sputter coater (Denton Vacuum, Inc., Moorestown NJ) to improve the conductivity of the samples and the quality of the SEM images. 

## Supporting Information

File S1 **Tables S1, S2 and S3** Table S1 in File S1, Primers used in this study. Table S2 in File S1, Sugar components in the time course hydrolysis of Avicel, Filter paper, and PASC by CbCel9A/Cel48A wild-type (WT) and its truncational mutants (TM1, TM2 and TM3). Table S3 in File S1, Sugar components in the time course hydrolysis of cellotetraose (G4) and cellopentaose (G5) by CbCel9A/Cel48A-WT and its truncational mutants (TM1, TM2 and TM3).
(DOCX)

File S2 **Figure S1, S2 and S3** Figure S1 in File S2, SDS-PAGE analysis of CbCel9A/Cel48A-WT and its truncation mutants (TM1, TM2 and TM3). M: protein molecular weight marker. Two µg of each enzyme was loaded on a 12% SDS polyacrylamide gel and stained with Coomasie Blue. Figure S2 in File S2, Optimum pH analysis of CbCel9A/Cel48A-WT and its truncation mutants (TM1 and TM2). The pH optimization of CbCel9A/Cel48AWT and its truncational mutants (TM1 and TM2) were determined by incubating the enzymes (400 nM for WT and TM1 and 600 nM for TM2) with PASC (1 mg ml-1) for 1 hour in a citrate buffer ranging from pH 3.0 to pH 6.0 and in a phosphate buffer ranging from pH5.5 to pH8.0 at 75°C. Figure S3 in File S2, Thermostability of CbCel9A/Cel48A Wild-type (WT) and its truncation mutants. A: CbCel9A/Cel48A wild-type; B: CbCel9A/Cel48A TM1; C: CbCel9A/Cel48A TM2; D: CbCel9A/Cel48A TM3; E: CbCel9A/Cel48A TM4. The enzymes were incubated at 70°C, 75°C, and 80°C (WT, TM1, TM2, TM3, and TM4) on a Veriti 96-well thermal cycler. Samples were taken at different time points and measured for residual activity using PASC as the substrate.
(DOCX)

File S3 **Figure S4, S5 and S6** Figure S4 in File S3, Alignment of the GH48 catalytic modules from CbCel9A/Cel48A, Clostridium cellulolyticum CcCel48F, and Thermobifida fusca TfCel48A. According to the crystal structures of CcCel48F (1G9J, 1G9G, and 2QNO), residues marked by an asterisk are candidate acid/base residues in the hydrolysis reaction; residues marked by the inverted open triangle may be important in the interaction of the protein with cello-oligosaccharide; residues marked by the inverted filled triangle may form hydrogen-bonds with cello-oligosaccharide. Residue marked by rectangles may have flexible contact in sugar transport. Figure S5 in File S3, A model three-dimensional structure of the GH48 catalytic module of CbCel9A/Cel48A. The three-dimensional structure 1G9J of Clostridium cellulolyticum CcCel48F was used as the template. Residues colored in blue are long hemithiocello-oligosaccharides. Figure S6 in File S3, Hydrolysis of Avicel, Filter paper and PASC by CbCel9A/Cel48A wild-type (WT), its truncational mutants, and their binary combinations as analyzed by an HPLC method. The experiments were carried out in citrate buffer (pH6.0) at 75°C for 16 h, by incubating 0.4 µM of each enzyme with 10 mg ml-1 of Avicel or with 16 discs of Whatman No. 1 filter paper (0.6 cm in diameter) or by incubating 0.2 µM of each enzyme with 2.5 mg ml-1 of PASC as the substrate.
(DOCX)

File S4 **Figure S7, S8 and S9** Figure S7 in File S4, The surface appearance of Avicel with enzyme treatment (WT, TM1, TM2 and TM3) and without (control). SEM images under 40,000x magnification. 0.4 µM of CbCel9A/Cel48A wild-type or the truncation mutants were incubated with 10 mg ml-1 of Avicel in citrate buffer (pH6.0) at 75°C for 24 hours. Figure S8 in File S4, Schematic representation of cello-oligosaccharide and crystalline cellulose degradation by GH9 and GH48. A: Hydrolysis of cello-oligosaccharides (G5) by GH9/CBM3c and GH48. B: Synergistic hydrolysis of crystalline cellulose by GH9/CBM3c, CBM3bs and GH48. New ends are generated by binding of the GH48 catalytic module and the CBM3bs to crystalline cellulose. Both the new and old ends are then attacked by the GH9/CBM3c to generate shorter products. Figure S9 in File S4, Synergistic effects of CbCel9A/Cel48A wild-type or its TM2 mutant in combination with four endoglucanases from C. bescii in cellulosic substrate hydrolysis and analysis of end products by an HPLC method. 4EGs: a mixture of four recombinant endoglucanses from C. bescii (CbMan5B/Cel44A-TM2, CbCel9B/Man5A-TM1, CbMan5C/Cel5A-TM2, and CbCel5B-TM1). The reactions were carried out in citrate buffer (pH5.5) at 70 °C for 16 h, by incubation of different concentrations (0, 0.5, 1.0, and 2.0 µM) of WT or TM2 with 5 mg ml-1 of Avicel, 5 mg ml-1 of PASC, or 10 mg ml-1 of Miscanthus, in the presence of the four endoglucanases (4EGs, each at 0.5 µM).
(DOCX)
