# Supplementary material for: Molecular and Biochemical Analyses of CbCel9A/Cel48A, a Highly Secreted Multi-Modular Cellulase by Caldicellulosiruptor bescii during Growth on Crystalline Cellulose
Source: PLoS One. 2013 Dec 16;8(12):e84172. doi: 10.1371/journal.pone.0084172 (PMC3865294; doi:10.1371/journal.pone.0084172)
Supplement: File S1 — Tables S1, S2 and S3 Table S1 in File S1, Primers used in this study. Table S2 in File S1, Sugar components in the time course hydrolysis of Avicel, Filter paper, and PASC by CbCel9A/Cel48A wild-type (WT) and its truncational mutants (TM1, TM2 and TM3). Table S3 in File S1, Sugar components in the time course hydrolysis of cellotetraose (G4) and cellopentaose (G5) by CbCel9A/Cel48A-WT and its truncational mutants (TM1, TM2 and TM3). (DOCX) [file pone.0084172.s001.docx]

**Supplemental Table S1**. **Primers used in this study.**

| Protein | Orientation | Sequence (5'-3')^a^ |
| --- | --- | --- |
| WT^b^ | Forward | AGATCTGGCAAGAGGTTAGGGCTGGTTCGTTTAAC |
|  | Reverse | GGTACCATTGATTGCCAAACAGTATTTCATATGTTGC |
| TM1^c^ | Forward | GACGACGACAAGATGCAAGAGGTTAGGGCTGGTTCGTTTAAC |
|  | Reverse | GAGGAGAAGCCCGGTTACCACATAAACCTCTGCCCATATTCGCC |
| TM2^b^ | Forward | AGATCTTCTTTGTTGAAGCTGGTATAAATGC |
|  | Reverse | GGTACCATTGATTGCCAAACAGTATTTCATATGTTGC |
| TM3^c^ | Forward | GACGACGACAAGATGCAAGAGGTTAGGGCTGGTTCGTTTAAC |
|  | Reverse | GAGGAGAAGCCCGGTTATACCTTTATCTGTCCACCTGCTACAGG |
| TM4^c^ | Forward | GACGACGACAAGATGCAAGAGGTTAGGGCTGGTTCGTTTAAC |
|  | Reverse | GAGGAGAAGCCCGGTTACAATTGATACATCTTTGCTAATGCACCCAC |
| TM4/  W775A^d^ | Forward | CAGAGTGCGATATCAGACGCGGCACAGATAGGAGCAAG |
| TM4/  W830A^d^ | Forward | GAGATACAGATAAGGTTTAACAAGAGTGATGCGAGCAATTACAATCAGG |

^a^: The underlined nucleotides are restriction enzyme recognition sites.

^b^: For expression in *B. megaterium.*

^c^: For expression in *E. coli.*

^d^: Site mutantion of TM4.

**Supplemental Table S2.** **Sugar components in the time course hydrolysis of Avicel, Filter paper, and PASC by CbCel9A/Cel48A wild-type (WT) and its truncational mutants (TM1, TM2 and TM3).**

| Substrate | Time (h) | WT | | |  | TM1 | | |  | TM2 | | |  | TM3 | | |
| --- | --- | --- | --- | --- | --- | --- | --- | --- | --- | --- | --- | --- | --- | --- | --- | --- |
|  |  | G1 (mM) | G2 (mM) | G3 (mM) |  | G1 (mM) | G2 (mM) | G3 (mM) |  | G1 (mM) | G2 (mM) | G3 (mM) |  | G1 (mM) | G2 (mM) | G3 (mM) |
|  |  |  |  |  |  |  |  |  |  |  |  |  |  |  |  |  |
| Avicel | 1 | 0.2±0.0 | 0.1±0.0 | 0.1±0.0 |  | <0.1 | <0.1 | 0.1±0.0 |  | ND | <0.1 | ND |  | 0.1±0.0 | <0.1 | <0.1 |
|  | 2 | 0.3±0.1 | 0.2±0.1 | 0.2±0.1 |  | 0.1±0.0 | <0.1 | 0.2±0.0 |  | ND | <0.1 | <0.1 |  | 0.1±0.0 | <0.1 | <0.1 |
|  | 4 | 0.5±0.2 | 0.3±0.1 | 0.3±0.1 |  | 0.2±0.0 | 0.1±0.0 | 0.2±0.0 |  | ND | <0.1 | <0.1 |  | 0.1±0.0 | <0.1 | <0.1 |
|  | 24 | 1.4±0.2 | 0.9±0.2 | 0.1±0.0 |  | 0.5±0.1 | 0.4±0.0 | 0.3±0.1 |  | ND | 0.2±0.0 | <0.1 |  | 0.2±0.0 | <0.1 | <0.1 |
|  |  |  |  |  |  |  |  |  |  |  |  |  |  |  |  |  |
| Filter paper | 1 | 0.2±0.0 | <0.1 | <0.1 |  | 0.1±0.0 | <0.1 | 0.1±0.0 |  | ND | ND | ND |  | 0.2±0.0 | <0.1 | <0.1 |
|  | 2 | 0.3±0.1 | 0.1±0.1 | 0.1±0.1 |  | 0.3±0.0 | 0.2±0.0 | 0.2±0.1 |  | ND | ND | ND |  | 0.2±0.1 | <0.1 | 0.1±0.0 |
|  | 4 | 0.5±0.2 | 0.4±0.1 | 0.2±0.1 |  | 0.4±0.1 | 0.2±0.0 | 0.3±0.0 |  | ND | ND | ND |  | 0.3±0.1 | 0.1±0.1 | 0.2±0.1 |
|  | 24 | 1.5±0.7 | 1.3±0.6 | 0.1±0.0 |  | 1.0±0.1 | 0.7±0.1 | 0.2±0.0 |  | ND | <0.1 | ND |  | 0.5±0.0 | 0.2±0.0 | 0.2±0.0 |
|  |  |  |  |  |  |  |  |  |  |  |  |  |  |  |  |  |
| PASC | 0.25 | 0.1±0.0 | <0.1 | <0.1 |  | 0.1±0.0 | <0.1 | 0.2±0.0 |  | ND | <0.1 | ND |  | 0.2±0.0 | <0.1 | 0.1±0.0 |
|  | 0.5 | 0.2±0.1 | 0.1±0.0 | 0.2±0.1 |  | 0.2±0.0 | 0.1±0.0 | 0.3±0.1 |  | ND | <0.1 | <0.1 |  | 0.3±0.0 | 0.1±0.0 | 0.2±0.0 |
|  | 1 | 0.5±0.0 | 0.3±0.0 | 0.4±0.0 |  | 0.2±0.0 | 0.1±0.0 | 0.3±0.1 |  | ND | <0.1 | <0.1 |  | 0.4±0.0 | 0.2±0.0 | 0.3±0.0 |
|  | 2 | 0.5±0.1 | 0.3±0.1 | 0.4±0.1 |  | 0.4±0.0 | 0.2±0.0 | 0.5±0.0 |  | ND | <0.1 | <0.1 |  | 0.5±0.0 | 0.2±0.0 | 0.4±0.0 |
|  | 4 | 0.7±0.0 | 0.4±0.1 | 0.6±0.0 |  | 0.5±0.0 | 0.3±0.0 | 0.6±0.0 |  | ND | <0.1 | <0.1 |  | 0.7±0.0 | 0.3±0.0 | 0.5±0.0 |
|  | 24 | 1.0±0.0 | 0.6±0.1 | 0.6±0.0 |  | 0.9±0.1 | 0.5±0.1 | 0.9±0.1 |  | ND | <0.1 | <0.1 |  | 0.9±0.0 | 0.4±0.1 | 0.5±0.0 |

ND: not detected.

**Supplemental Table S3. Sugar components in the time course hydrolysis of cellotetraose (G4) and cellopentaose (G5) by CbCel9A/Cel48A-WT and its truncational mutants (TM1, TM2 and TM3).**

| Substrates | Time |  |  | WT |  |  |  |  | TM1 |  |  |  |  | | TM2 |  |  |  |  | TM3 |  |  |
| --- | --- | --- | --- | --- | --- | --- | --- | --- | --- | --- | --- | --- | --- | --- | --- | --- | --- | --- | --- | --- | --- | --- |
|  |  | G1 (mM) | G2  (mM) | G3  (mM) | G4  (mM) | G5  (mM) | G1 (mM) | G2  (mM) | G3  (mM) | G4  (mM) | G5  (mM) | G1 (mM) | G2  (mM) | G3  (mM) | | G4  (mM) | G5  (mM) | G1 (mM) | G2  (mM) | G3  (mM) | G4  (mM) | G5  (mM) |
| G4 | 2min | ND | <0.1 | ND | 3.8±0.1 | ND | <0.1 | 0.1±0.0 | 0.1±0.0 | 3.4±0.1 | ND | ND | ND | ND | | 3.6±0.2 | ND | ND | <0.1 | ND | 3.8±0.0 | ND |
|  | 10min | 0.1±0.1 | 0.2±0.1 | 0.2±0.1 | 3.7±0.5 | ND | 0.1±0.0 | 0.2±0.0 | 0.3±0.0 | 3.5±0.0 | ND | ND | ND | ND | | 3.8±0.6 | ND | 0.1±0.1 | 0.1±0.0 | 0.2±0.0 | 3.6±0.1 | ND |
|  | 2h | 0.8±0.1 | 0.8±0.2 | 1.5±0.0 | 1.1±0.5 | ND | 0.4±0.1 | 0.6±0.1 | 0.8±0.1 | 2.3±0.2 | ND | ND | <0.1 | ND | | 3.7±0.6 | ND | 0.7±0.0 | 1.0±0.0 | 1.1±0.0 | 1.4±0.2 | ND |
|  | 24h | 1.3±0.0 | 1.1±0.2 | 1.4±0.3 | ND | ND | 1.2±0.0 | 1.6±0.0 | 1.9±0.0 | <0.1 | ND | ND | 0.2±0.1 | ND | | 3.5±0.4 | ND | 1.4±0.6 | 1.4±0.2 | 1.5±0.4 | ND | ND |
|  |  |  |  |  |  |  |  |  |  |  |  |  |  |  | |  |  |  |  |  |  |  |
| G5 | 2min | 0.5±0.1 | 0.3±0.1 | 0.4±0.1 | 0.9±0.1 | 1.4±0.0 | 0.7±0.1 | 0.4±0.0 | 0.6±0.1 | 1.2±0.1 | 0.6±0.1 | ND | ND | ND | | ND | 2.1±0.0 | 0.3±0.0 | 0.2±0.0 | 0.2±0.0 | 0.5±0.0 | 1.7±0.0 |
|  | 10min | 1.0±0.1 | 0.5±0.1 | 0.7±0.1 | 1.6±0.3 | 0.5±0.1 | 0.9±0.1 | 0.4±0.0 | 0.6±0.0 | 1.4±0.1 | 0.3±0.1 | ND | ND | ND | | ND | 2.5±0.3 | 0.9±0.0 | 0.5±0.0 | 0.6±0.1 | 1.5±0.1 | 1.0±0.2 |
|  | 2h | 1.5±0.2 | 1.0±0.2 | 1.3±0.3 | 1.1±0.2 | ND | 1.0±0.1 | 0.7±0.1 | 1.0±0.1 | 1.0±0.0 | ND | ND | <0.1 | <0.1 | | ND | 2.2±0.4 | 1.5±0.1 | 1.1±0.1 | 1.3±0.1 | 1.2±0.2 | ND |
|  | 24h | 1.4±0.2 | 1.2±0.1 | 1.6±0.1 | ND | ND | 1.3±0.0 | 1.0±0.0 | 1.3±0.0 | 0.1±0.0 | ND | ND | 0.2±0.2 | 0.3±0.1 | | ND | 1.6±0.1 | 1.6±0.1 | 1.3±0.2 | 1.4±0.1 | ND | ND |

ND: not detected.
