# Supplementary figures and images for: Molecular and Biochemical Analyses of CbCel9A/Cel48A, a Highly Secreted Multi-Modular Cellulase by Caldicellulosiruptor bescii during Growth on Crystalline Cellulose
Source: PLoS One. 2013 Dec 16;8(12):e84172. doi: 10.1371/journal.pone.0084172 (PMC3865294; doi:10.1371/journal.pone.0084172)

**Supplemental Figure S1.**


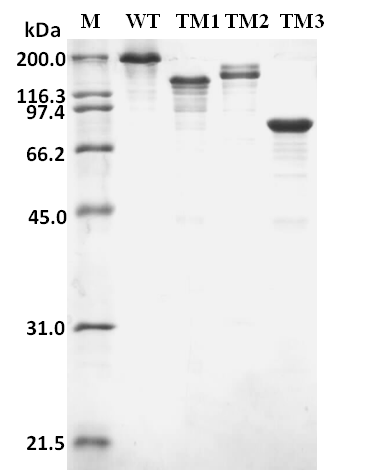


**Supplemental Figure S2**

**
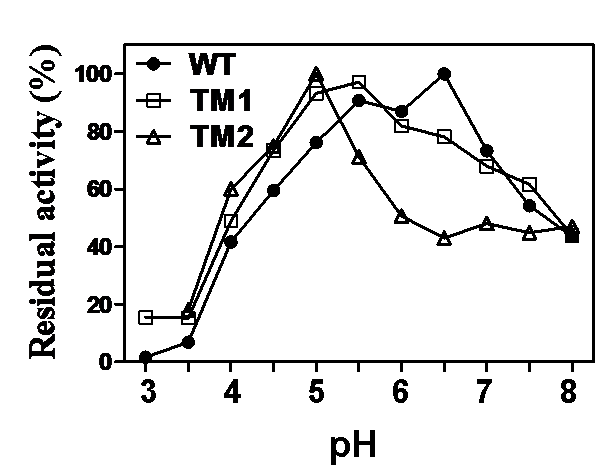
**

**Supplemental Figure S3**

**
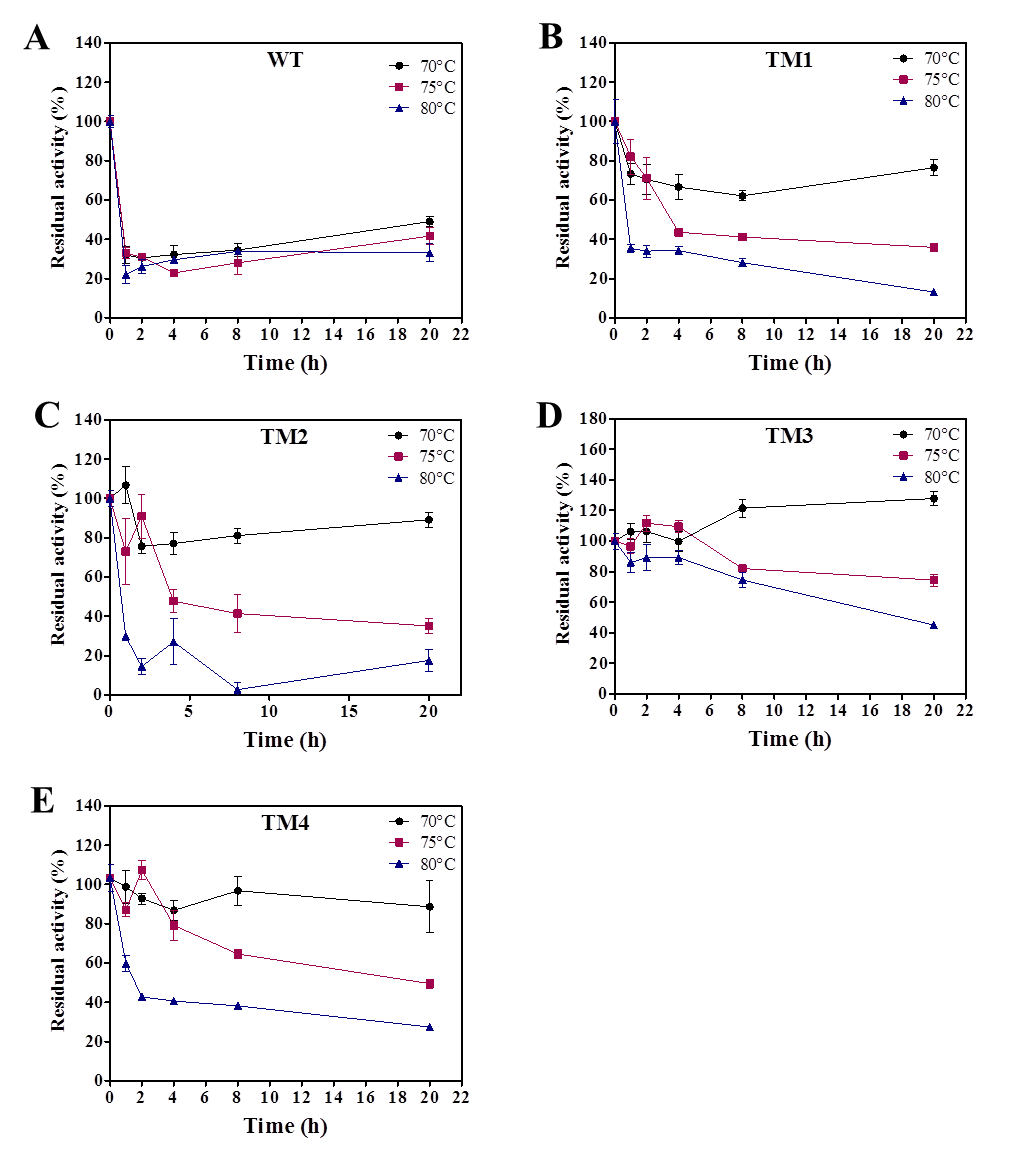
**

Supplement: File S2 — Figure S1, S2 and S3 Figure S1 in File S2, SDS-PAGE analysis of CbCel9A/Cel48A-WT and its truncation mutants (TM1, TM2 and TM3). M: protein molecular weight marker. Two µg of each enzyme was loaded on a 12% SDS polyacrylamide gel and stained with Coomasie Blue. Figure S2 in File S2, Optimum pH analysis of CbCel9A/Cel48A-WT and its truncation mutants (TM1 and TM2). The pH optimization of CbCel9A/Cel48AWT and its truncational mutants (TM1 and TM2) were determined by incubating the enzymes (400 nM for WT and TM1 and 600 nM for TM2) with PASC (1 mg ml-1) for 1 hour in a citrate buffer ranging from pH 3.0 to pH 6.0 and in a phosphate buffer ranging from pH5.5 to pH8.0 at 75°C. Figure S3 in File S2, Thermostability of CbCel9A/Cel48A Wild-type (WT) and its truncation mutants. A: CbCel9A/Cel48A wild-type; B: CbCel9A/Cel48A TM1; C: CbCel9A/Cel48A TM2; D: CbCel9A/Cel48A TM3; E: CbCel9A/Cel48A TM4. The enzymes were incubated at 70°C, 75°C, and 80°C (WT, TM1, TM2, TM3, and TM4) on a Veriti 96-well thermal cycler. Samples were taken at different time points and measured for residual activity using PASC as the substrate. (DOCX) [file pone.0084172.s002.docx]

**Supplemental Figure S4**


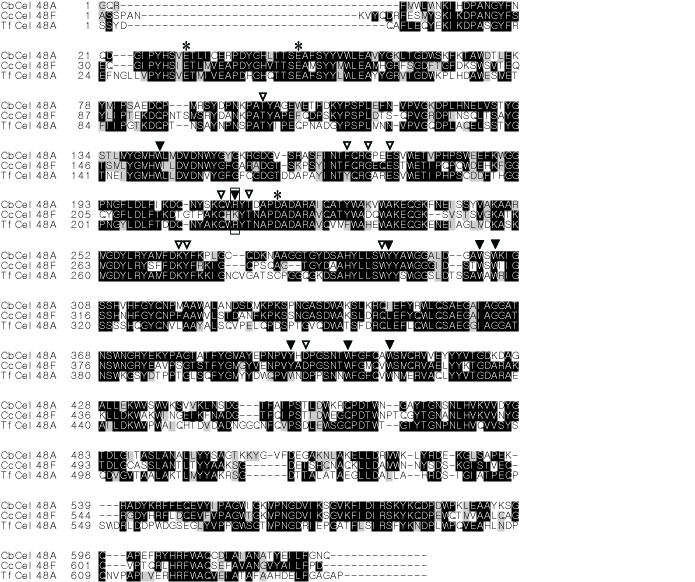


**Supplemental Figure S5**


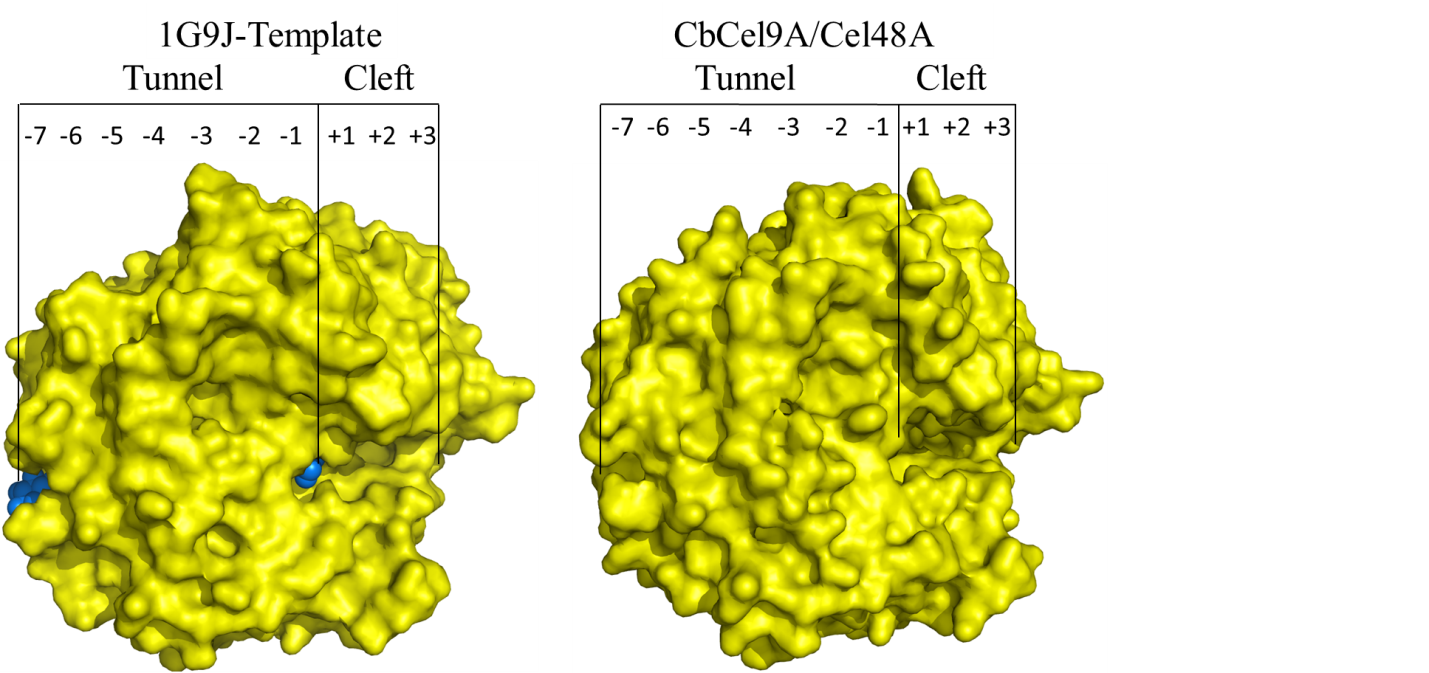


**Supplemental Figure S6**


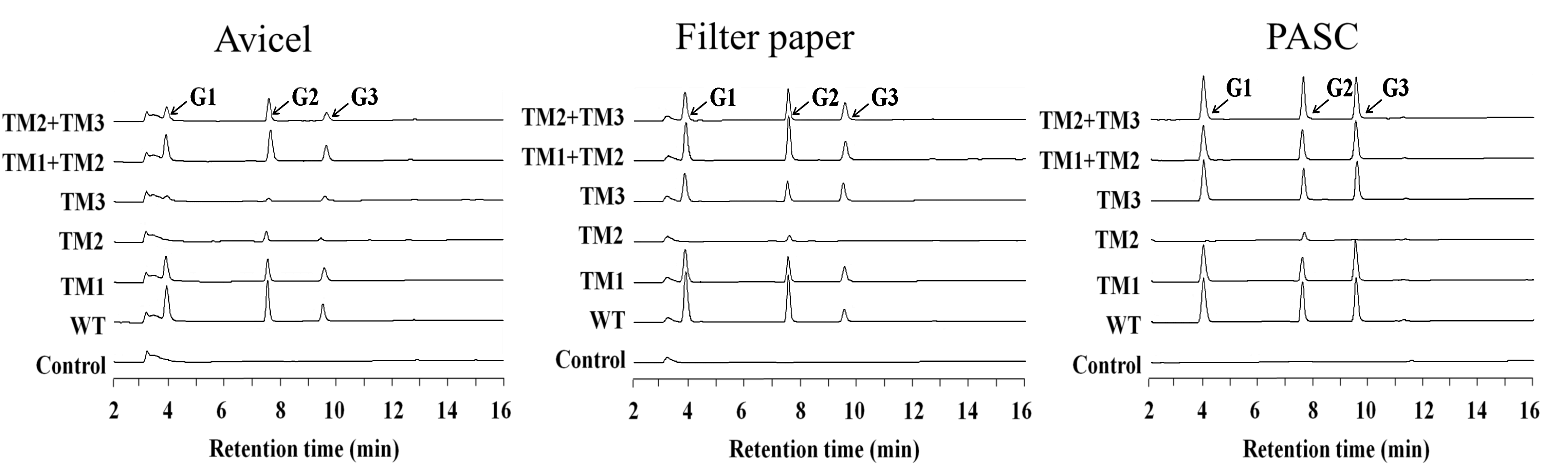

Supplement: File S3 — Figure S4, S5 and S6 Figure S4 in File S3, Alignment of the GH48 catalytic modules from CbCel9A/Cel48A, Clostridium cellulolyticum CcCel48F, and Thermobifida fusca TfCel48A. According to the crystal structures of CcCel48F (1G9J, 1G9G, and 2QNO), residues marked by an asterisk are candidate acid/base residues in the hydrolysis reaction; residues marked by the inverted open triangle may be important in the interaction of the protein with cello-oligosaccharide; residues marked by the inverted filled triangle may form hydrogen-bonds with cello-oligosaccharide. Residue marked by rectangles may have flexible contact in sugar transport. Figure S5 in File S3, A model three-dimensional structure of the GH48 catalytic module of CbCel9A/Cel48A. The three-dimensional structure 1G9J of Clostridium cellulolyticum CcCel48F was used as the template. Residues colored in blue are long hemithiocello-oligosaccharides. Figure S6 in File S3, Hydrolysis of Avicel, Filter paper and PASC by CbCel9A/Cel48A wild-type (WT), its truncational mutants, and their binary combinations as analyzed by an HPLC method. The experiments were carried out in citrate buffer (pH6.0) at 75°C for 16 h, by incubating 0.4 µM of each enzyme with 10 mg ml-1 of Avicel or with 16 discs of Whatman No. 1 filter paper (0.6 cm in diameter) or by incubating 0.2 µM of each enzyme with 2.5 mg ml-1 of PASC as the substrate. (DOCX) [file pone.0084172.s003.docx]

**Supplemental Figure S7**


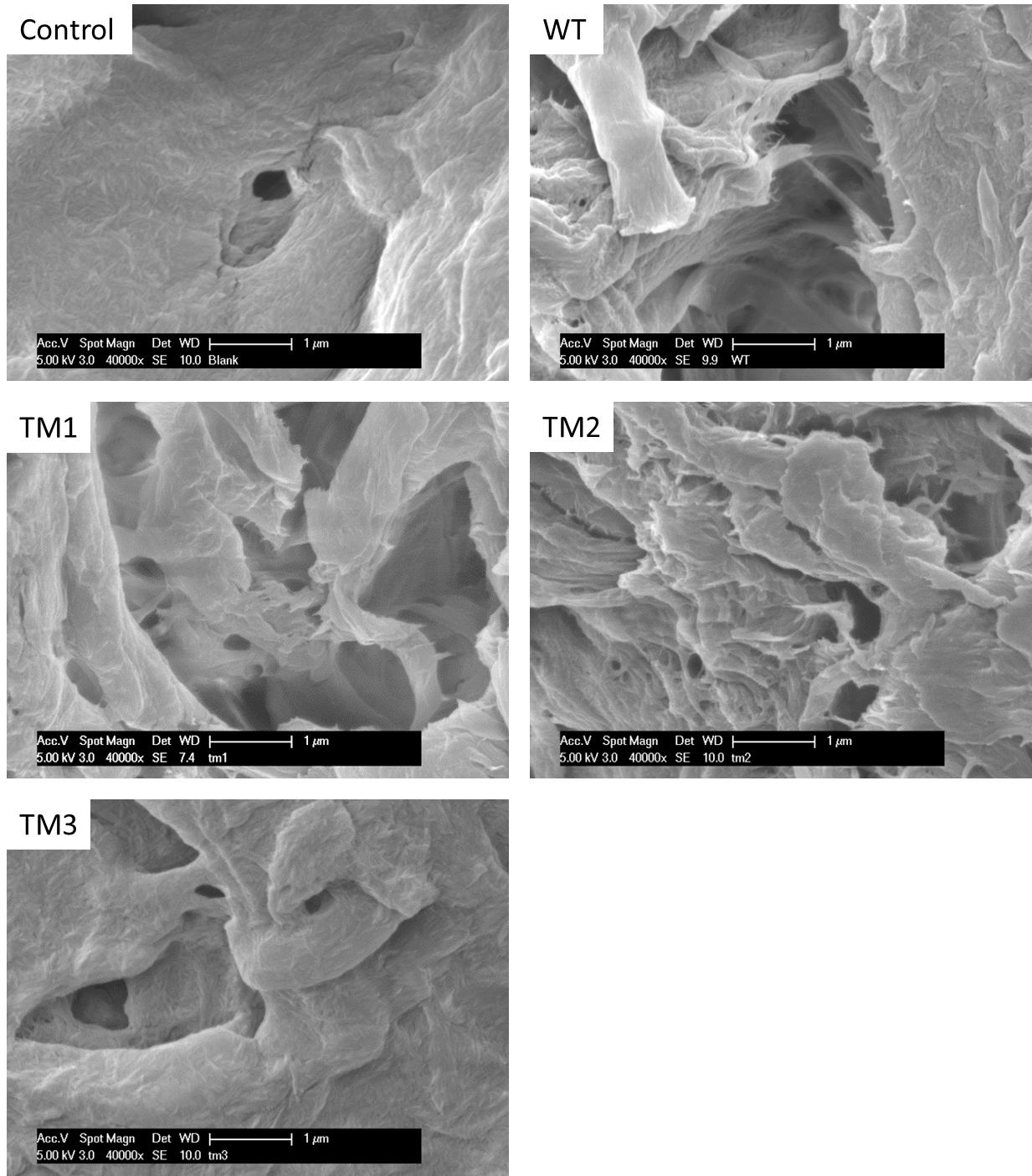


**Supplemental Figure S8**


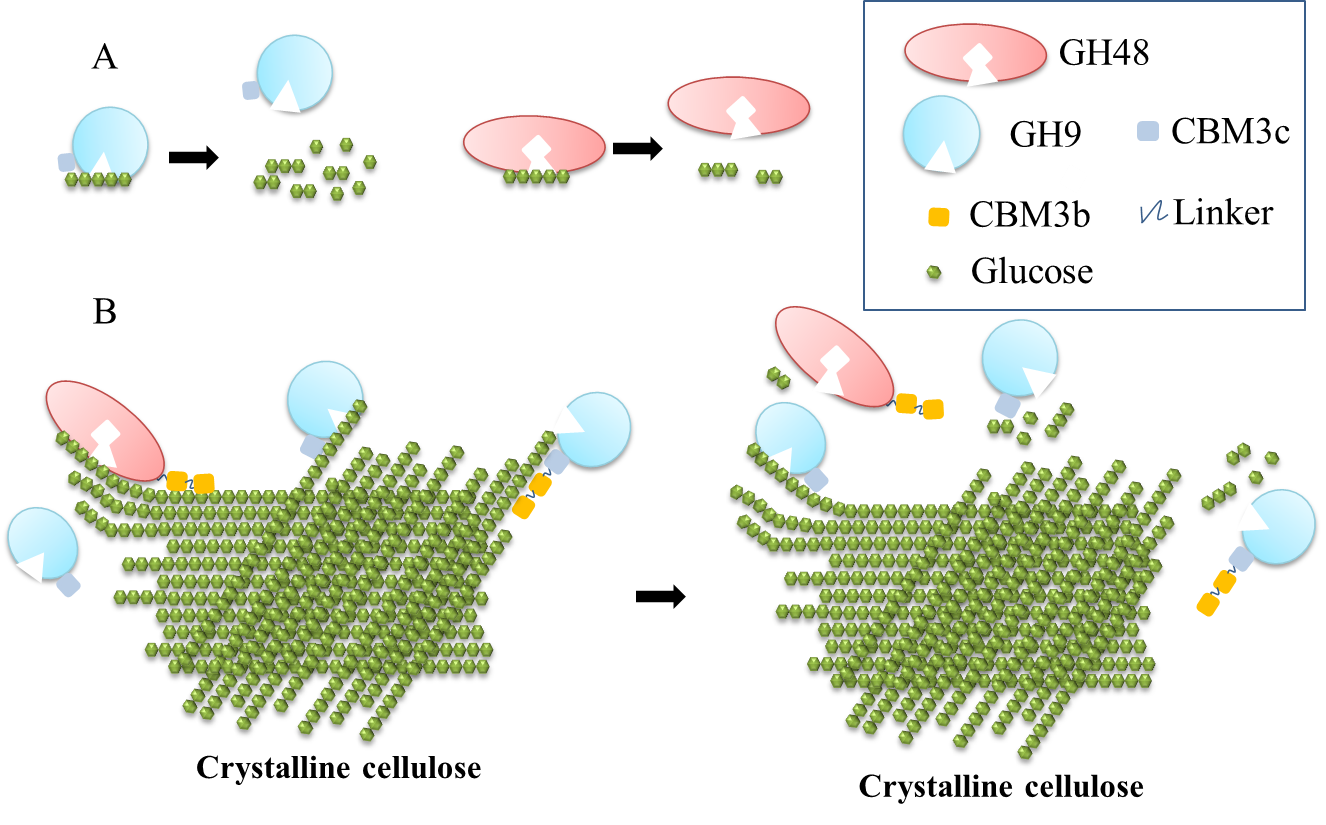


**Supplemental Figure S9**


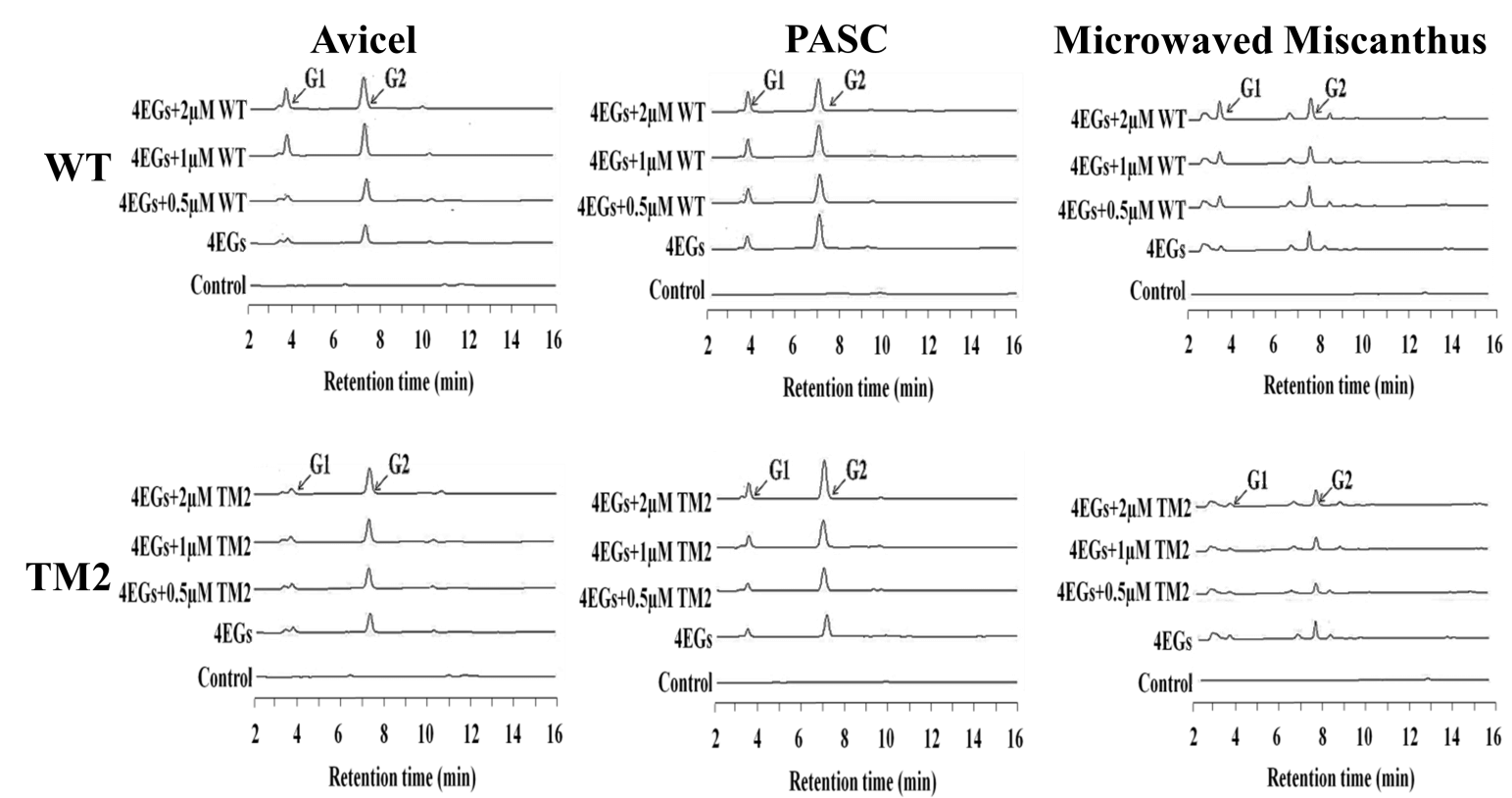

Supplement: File S4 — Figure S7, S8 and S9 Figure S7 in File S4, The surface appearance of Avicel with enzyme treatment (WT, TM1, TM2 and TM3) and without (control). SEM images under 40,000x magnification. 0.4 µM of CbCel9A/Cel48A wild-type or the truncation mutants were incubated with 10 mg ml-1 of Avicel in citrate buffer (pH6.0) at 75°C for 24 hours. Figure S8 in File S4, Schematic representation of cello-oligosaccharide and crystalline cellulose degradation by GH9 and GH48. A: Hydrolysis of cello-oligosaccharides (G5) by GH9/CBM3c and GH48. B: Synergistic hydrolysis of crystalline cellulose by GH9/CBM3c, CBM3bs and GH48. New ends are generated by binding of the GH48 catalytic module and the CBM3bs to crystalline cellulose. Both the new and old ends are then attacked by the GH9/CBM3c to generate shorter products. Figure S9 in File S4, Synergistic effects of CbCel9A/Cel48A wild-type or its TM2 mutant in combination with four endoglucanases from C. bescii in cellulosic substrate hydrolysis and analysis of end products by an HPLC method. 4EGs: a mixture of four recombinant endoglucanses from C. bescii (CbMan5B/Cel44A-TM2, CbCel9B/Man5A-TM1, CbMan5C/Cel5A-TM2, and CbCel5B-TM1). The reactions were carried out in citrate buffer (pH5.5) at 70 °C for 16 h, by incubation of different concentrations (0, 0.5, 1.0, and 2.0 µM) of WT or TM2 with 5 mg ml-1 of Avicel, 5 mg ml-1 of PASC, or 10 mg ml-1 of Miscanthus, in the presence of the four endoglucanases (4EGs, each at 0.5 µM). (DOCX) [file pone.0084172.s004.docx]
